# Basin-scale biogeography of marine phytoplankton reflects cellular-scale optimization of metabolism and physiology

**DOI:** 10.1126/sciadv.abl4930

**Published:** 2022-01-21

**Authors:** John R. Casey, Rene M. Boiteau, Martin K. M. Engqvist, Zoe V. Finkel, Gang Li, Justin Liefer, Christian L. Müller, Nathalie Muñoz, Michael J. Follows

**Affiliations:** 1 Department of Earth, Atmospheric, and Planetary Sciences, Massachusetts Institute of Technology, Cambridge, MA, USA.; 2 School of Ocean and Earth Science and Technology, University of Hawai‘i at M$\bar{a}$noa, Honolulu, HI, USA.; 3 College of Earth, Ocean, and Atmospheric Sciences, Oregon State University, Corvallis, OR, USA.; 4 Department of Biology and Biological Engineering, Chalmers University of Technology, Göteborg, Sweden.; 5 Department of Oceanography, Dalhousie University, Halifax, NS, Canada.; 6 Department of Biology, Mount Allison University, Sackville, NB, Canada.; 7 Center for Computational Mathematics, Flatiron Institute, New York, NY, USA.; 8 Environmental Molecular Sciences Division, Pacific Northwest National Laboratories, Richland, WA, USA.

## Abstract

Extensive microdiversity within *Prochlorococcus*, the most abundant marine cyanobacterium, occurs at scales from a single droplet of seawater to ocean basins. To interpret the structuring role of variations in genetic potential, as well as metabolic and physiological acclimation, we developed a mechanistic constraint-based modeling framework that incorporates the full suite of genes, proteins, metabolic reactions, pigments, and biochemical compositions of 69 sequenced isolates spanning the *Prochlorococcus* pangenome. Optimizing each strain to the local, observed physical and chemical environment along an Atlantic Ocean transect, we predicted variations in strain-specific patterns of growth rate, metabolic configuration, and physiological state, defining subtle niche subspaces directly attributable to differences in their encoded metabolic potential. Predicted growth rates covaried with observed ecotype abundances, affirming their significance as a measure of fitness and inferring a nonlinear density dependence of mortality. Our study demonstrates the potential to interpret global-scale ecosystem organization in terms of cellular-scale processes.

## Teaser

Resolving the nano-niche: Simulations of metabolism and physiology across pangenomes link the molecular and ecosystem scales.

## INTRODUCTION

The cyanobacterium *Prochlorococcus* is the most abundant photosynthetic organism in the ocean, contributing most of the primary production in vast regions of the nutrient-starved subtropical gyres (*1*–*3*). The dominance of *Prochlorococcus* in these “ocean deserts” has been attributed to their small size (*4*), streamlined genomes (*5*), and their microdiversity (*6*, *7*). At the subspecies clade level, “ecotypes” of *Prochlorococcus* partition with depth in the water column and along temperature gradients to span the full euphotic layer from about 40°N to 40°S (*8*–*11*) and exhibit differential activity within the total *Prochlorococcus* assemblage (population, herein) (*12*). To maintain access to a low but variable supply of many organic and inorganic nutrient resources, it may be that *Prochlorococcus* shifts the costs of housing a broad metabolic repertoire from the individual cell to the population. While a typical strain contains fewer than 2000 protein-coding genes, the pangenome of *Prochlorococcus* may carry more than 80,000 protein-coding genes (*13*), a vast library of genetic potential distributed among a nonredundant, diverse minority. Extensive metagenomic surveys and single-cell sequencing have provided a glimpse into the scope of the pangenome (*14*) and the co-occurrence of many strains (*6*, *12*), but it is unclear how metabolic and physiological microdiversity influences the *Prochlorococcus* population fitness across the ocean.

*Prochlorococcus* populations make use of a broad repertoire of strategies to respond to changes in nutrient availability and light conditions through its genetic diversity (*15*), photoadaptation and photoacclimation (*16*, *17*), flexibility in nutrient uptake kinetics (*18*), and changes in elemental stoichiometry (*19*, *20*). Although much has been learned about the biology of *Prochlorococcus*, its physiology, genetic diversity, and adaptations to its spartan lifestyle, the current generation of statistical and dynamic models used to interpret and predict *Prochloroccus* biogeography and biogeochemistry (*21*–*23*) reflects little of the depth of diversity or current mechanistic understanding. Drawing from extensive genomic and molecular datasets, we present a model that spans the genetic microdiversity of *Prochlorococcus* and resolves physiological and metabolic acclimation to local environments. It can provide a framework to simulate and interpret the essential diversity and flexibility of this organism and the organization of its populations at the ecosystem scale.

## RESULTS AND DISCUSSION

### The Microbial Simulation Environment

**Fig. 1.**
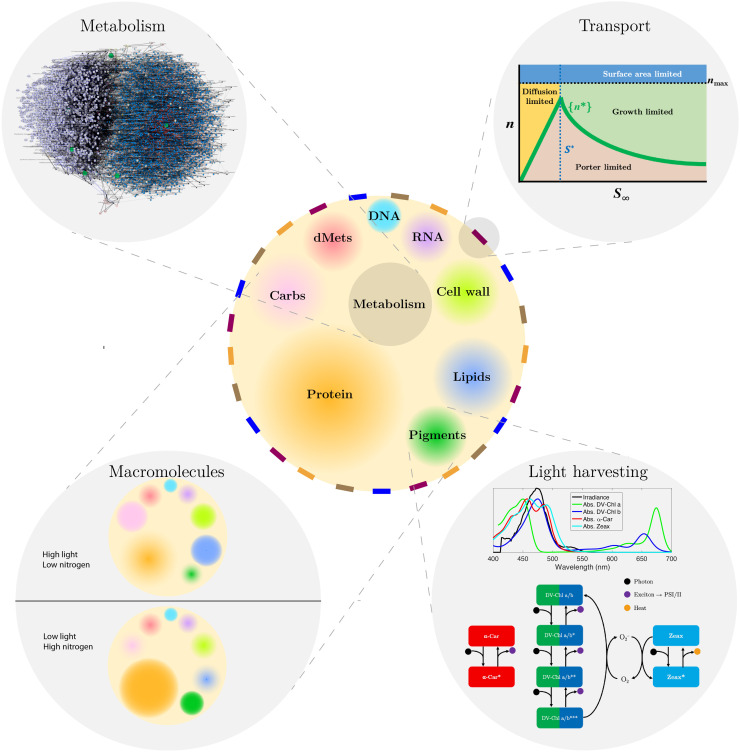
Schematic of metabolic and physiological acclimation processes simulated by MSE. A single cell is shown at the center with several membrane transporters (colored rectangles) and macromolecular pools (graded circles). Four key modules are shown in expanded detail, described clockwise. (**Top left**) Metabolic networks differ for each of the 69 strains simulated. A multipartite graph of genes, reactions, and metabolites is shown for a particular strain, SS120. (**Top right**) Transporter abundances (*n*) are optimized according to a mechanistic model of substrate transport that resolves several different limitation regimes (diffusion limitation, growth limitation, porter limitation, and surface area limitation). Competition for resources between transporters results in deviations from the optimum n^*^ across the range of ambient substrate concentrations *S*_∞_. (**Bottom right**) The downwelling irradiance spectrum selects the optimal distribution of pigments on the basis of absorption (Abs) spectra and the cellular energy demand, within experimental constraints. Excitons are shuttled to photosystems I and II from each electronic state of each light-harvesting pigment [divinylchlorophyll a (DV-Chl a), divinylchlorophyll b (DV-Chl b), and α-carotene (α-Car)]. Under excess light conditions, the photoprotective pigment zeaxanthin (Zeax) acts both to dissipate excess photons from light-harvesting pigments to heat and to quench singlet oxygen generated by the relaxation of triply excited chlorophylls a and b. (**Bottom left**) Macromolecular compositions are optimized within experimental constraints to maximize growth given the availability of nutrient and energy resources. Differences between environments in the size of each graded circle are intended to reflect changes in the levels of each macromolecular pool. Carbs, carbohydrates; dMets, dissolved (free) metabolites.

We developed the Microbial Simulation Environment (MSE; http://github.com/jrcasey/mse_AMT; Fig. 1), a pipeline to simulate the steady-state growth, metabolism, and physiology of both sequenced isolates and in silico strains, each acclimated to a particular environment. Briefly, a pangenome-scale metabolic model (PanGEM) was reconstructed, drawing from a database of 866,894 protein coding sequences across 647 sequenced isolates, metagenome-assembled genomes, and single cell–amplified genomes spanning the *Prochlorococcus* phylogeny (DOI:10.5281/zenodo.4477905; data S1). The PanGEM consists of 1117 orthologous gene clusters, 1484 reactions, and 1282 metabolites and was manually curated and annotated with multiple bioinformatics, cheminformatics, and systems biology databases and tools (Materials and Methods and table S1). On the basis of a power law regression (*24*) of pangenome rarefaction curves generated for both homologous genes and orthologous gene clusters annotated in the Kyoto Encyclopedia of Genes and Genomes (KEGG) (fig. S1), the genetic diversity remains open (α = 0.8) but the functionally annotated metabolic subset appears to be closed (α = 1.9), suggesting that much of the genetic diversity yet to be cataloged will be associated with regulatory, repair, and other nonmetabolic functions. Even so, gap filling of 36 reactions (2.4% of all reactions) lacking an annotated gene was still necessary for biomass synthesis in the PanGEM reconstruction, so additional functional annotation efforts are needed to provide evidence for these missing gene-protein-reaction associations.

Using the PanGEM as a template, feasible and stoichiometrically balanced strain-specific genome-scale models (GEMs) for each sequenced organism (referred to as in silico strains) are generated using a compressed sensing (CS)–based approach (PanGEM Toolbox; http://github.com/jrcasey/PanGem; Materials and Methods and fig. S2) and quality tested using a community standard (*25*). Before simulations, each in silico strain acclimates to a given environment (defined by in situ temperature, nutrient concentrations, and light spectra) by optimally adjusting the cell size, nutrient transporters, pigments, biochemical compositions, and metabolic fluxes, accomplished with a sequence of experimentally and empirically constrained bilevel flux balance analysis (FBA) optimizations (called OptTrans and PhysOpt; Materials and Methods). Metabolic rates are then adjusted for strain-specific temperature dependence, predicted from proteome sequences with an amended machine learning algorithm [based on (*26*)] (Materials and Methods). Together, PanGEM Toolbox and MSE are intended to be suitably flexible for implementation in other microbial systems and are documented to guide users through reconstruction and simulation.

**Fig. 2.**
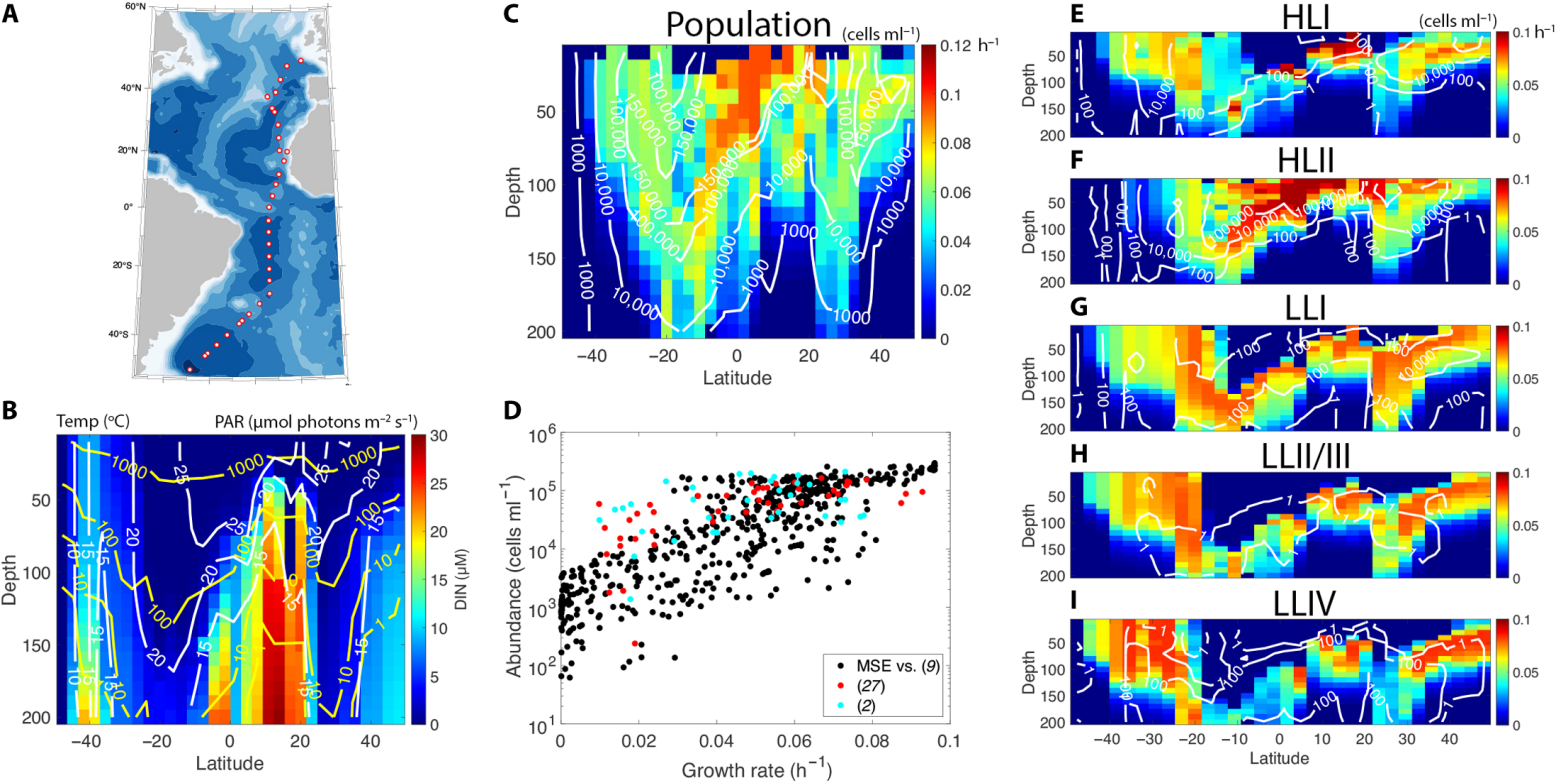
Summary of AMT-13 cruise simulation. (**A**) Cruise track of the AMT-13 expedition during the fall of 2003. (**B**) Meridional sections of observed temperature (white contours), photosynthetically active radiation PAR; (yellow contours, logarithmically spaced), and dissolved inorganic nitrogen (DIN; heatmap). (**C**) Meridional sections of observed *Prochlorococcus* abundances (white contours, logarithmically spaced) and predicted population growth rates (heatmap). (**D**) Observed *Prochlorococcus* population abundance plotted against predicted population growth rates (black markers). Red and blue markers correspond to previously published datasets. (**E** to **I**) Meridional sections of measured *Prochlorococcus* ecotype abundances (white contours, logarithmically spaced) and predicted growth rates (heatmaps). Although the LLII/III ecotype was nearly absent from the entire transect, Johnson *et al*. (*9*) note that quantitative polymerase chain reaction primers for this ecotype may have been contaminated.

MSE simulations were implemented for 69 sequenced isolates representing all five major ecotypes of *Prochlorococcus* (data S1) on the Atlantic Meridional Transect cruise (AMT-13) in late summer of 2003. A suite of 66 physical, chemical, and biological measurements from satellite optics, shipboard sensors, and discrete samples were interpolated at 10-m depth intervals from 10 to 200 m at 32 stations from 48°N to 45°S, passing through the Senegalo-Mauritanian upwelling region (Fig. 2A and data S2). A subset of variables including temperature, solar irradiance, and nutrient concentrations were used as inputs to each simulation, while other variables were used for context and validation (figs. S3 to S5).

Leveraging a synoptic dataset of *Prochlorococcus* ecotype abundances along the transect (*9*), predicted growth rates, metabolic rates, and biochemical compositions at the strain level were scaled up to populations. By assuming that each ecotype could be represented by its fittest strain in a particular sample location, the rates and compositions associated with each ecotype were weighted by their observed in situ abundances (Materials and Methods) to directly compare against measured, volumetric quantities.

### *Prochlorococcus* population ecology and biogeochemistry

The predicted vertical structure in total *Prochlorococcus* population growth rates (Fig. 2C and fig. S6) was consistent with observed abundance profiles (*2*, *27*), generally declining with depth and light intensity and in the top few meters because of photoinhibition and nutrient scarcity. While our approach considers only bottom-up controls, there was a remarkable correspondence at the population level between predicted growth rates and observed abundances throughout the transect (Fig. 2D). Overall, a nonlinear relationship emerged between observed abundances and predicted growth rates; a power law (of the form $\frac{\mathrm{dN}}{\mathrm{dt}}=\mu B-mB^{x}$) provided a better fit (*R*^2^ = 0.79; df = 638) than a linear form (*R*^2^ = 0.40), suggesting a nonlinear density dependence of mortality. Direct taxon-specific in situ phytoplankton growth rate measurements are scarce (*1*, *2*, *27*) but follow a qualitatively similar pattern (Fig. 2D).

Within the total *Prochlorococcus* population, the predicted growth rates of each ecotype showed distinct environmental niches that corresponded to observed abundances (Fig. 2, E to I; shading shows growth rate and contours show observed population density). This correspondence supports the notion that growth rate is important for relative fitness. Generally, growth of HLII ecotype strains outpaced others in the warmest environments where inorganic nitrogen concentrations were lowest and light was highest, while HLI strains were fittest in the midlatitudes. LLI and LLII/III ecotypes overlapped in their optimal niche space around the deep chlorophyll maximum depth, below which growth rates were typically highest for strains belonging to the larger, higher–gene content ecotype LLIV.

At high latitudes, the temperatures, nutrient concentrations, and light levels near the surface correspond to those typical of the deep chlorophyll maximum in the central gyres, resulting in an outcropping of low light–adapted ecotypes. In the northern hemisphere, LLI was the dominant ecotype at the surface, both in our simulations of growth rates and in observed abundance. Breaking the general trend of correspondence, we predicted high growth rates of low light–adapted ecotypes in the southernmost stations of the transect where the observed population density was low. This might reflect a dominant top-down control of populations in that region (*28*) that cannot be captured by the bottom-up growth model. It might also be noted that the AMT-13 transect was conducted during the late boreal summer and austral winter, and mixed layer depths at stations south of 22°S were greater than 140 m (based on the Δσ_t_ = 0.125 kg m^−3^ criterion). Hence, potentially important light history dynamics associated with deep convective mixing may be improperly captured by our steady-state model.

At stations where these direct comparisons were possible, the contribution of *Prochlorococcus* to total depth-integrated gross oxygen production (fig. S7) followed an expected trend, contributing less (15 ± 14%) in high-chlorophyll regions (>15 mg chlorophyll a m^−2^) and more (52 ± 31%) in the oligotrophic regions, in line with previous reports (*1*, *3*). Although a direct comparison was not possible, a subsurface maximum in the *f*-ratio (the proportion of nitrogen uptake supplied by nitrate) (*29*) was predicted along the top of the deep chlorophyll maximum near the equator and lower subtropics (fig. S8), a hypothesis with implications for the contribution of *Prochlorococcus* to the biological carbon pump and that could conceivably be tested in situ (*30*).

The elemental composition and energy content of phytoplankton constrain marine food webs and the cycles of carbon, nitrogen, phosphorus, and other bioelements (*31*). Predicted carbon-to-nitrogen molar ratios of *Prochlorococcus* populations varied from 5.4 to 6.8 along the meridional transect (fig. S9), with higher values associated with deeper or higher-latitude samples. This range and pattern is consistent with previous measurements of *Prochlorococcus* elemental stoichiometry in the Sargasso Sea (5.4 to 7.4) (*20*). The energy available for consumers (here quantified by the enthalpy of combustion of biomass) also varied with light (68% of the predicted variance), with dry biomass spanning the range from 27.4 KJ gDW^−1^ near the surface to 30.3 KJ gDW^−1^ below the euphotic depth (fig. S10). Population elemental stoichiometry and energy content reflect both the strain composition of each population and the phenotypic states of those strains that, in turn, reflect a great many underlying physiological processes, including alterations to macromolecular compositions and photoacclimation.

### Physiological and metabolic acclimation

Departures in environmental variables from ideal laboratory growth conditions were reflected in changes to optimal cell physiology and metabolic flux distributions. Acclimation strategies involved altering uptake kinetics through changes in transporter abundances and cell size, altering elemental stoichiometry and growth yields through changes in macromolecular compositions, and altering photosynthetic performance through changes in pigmentation and electron flow pathways. The effect of allowing for physiological acclimation can be appreciated by monitoring phenotypic changes that give rise to fitness gains. Compared with strains prevented from acclimating beyond their nutrient-replete batch culture phenotype, transporter acclimation increased growth rates by 43 ± 26% and macromolecular and photoacclimation provided further growth rate gains of 47 ± 25% (fig. S11). Especially under low-light conditions, physiological acclimation was necessary to acquire sufficient reductant to exceed nongrowth associated maintenance adenosine triphosphate (ATP) demands. For example, the threshold for net positive growth of a high light–adapted strain (MIT 9312) acclimated to ideal laboratory conditions required a minimum flux of 17.7 μmol photons m^−2^ s^−1^ but was able to acclimate to as low as 2.1 μmol photons m^−2^ s^−1^. Photoacclimation may therefore provide an explanation for HLII cells found deeper in the water column (*9*, *10*) than might be expected from laboratory isolates (*17*). Thus, in addition to providing fitness gains within the feasible growth niche of each strain, physiological acclimation effectively extends the depth horizon and geographic range for growth.

The abundances of key nutrient transporters (e.g., ammonia, nitrite, nitrate, and phosphate) are regulated by a trade-off between synthesis costs, available space on the membrane, and uptake rates. OptTrans explicitly accounts for these trade-offs using a mechanistic model that combines quantitative proteomics, molecular modeling, and FBA to predict the optimal abundance of each transporter and the optimal cell size (Materials and Methods) (*32*). For any modeled strain, optimal cell sizes increased in proportion with external resource concentrations, but average population cell sizes (fig. S12) were driven more by community composition than by acclimation processes alone, indicating the importance of cell size as an adaptive trait. Our model explicitly differentiates states of nutrient limitation: Diffusion limitation occurs when external substrate concentrations limit the flux toward the cell below demands, and membrane surface area limitation occurs when the space afforded to transporters is exhausted, resulting in suboptimal uptake. When external substrates exceed a critical concentration S*, uptake rates are held at the level demanded by some other limiting resource (e.g., another limiting nutrient or the maximum growth rate). As expected, diffusion limitation was prevalent for nitrite and nitrate transport in the lower latitudes above the nutricline, where concentrations were routinely in the low-nanomolar range. Observed ammonium concentrations were predominantly just below the predicted S* (fig. S13), and transport was often limited by membrane surface area. It is possible that additional nitrogen sources that were not resolved in our simulation, especially urea and amino acids, supply the remaining deficit in nitrogen demanded by maximal growth rates; however, the competition for membrane space among multiple transporters would be even more prevalent. Phosphate concentrations were mostly above S^*^ throughout the transect, with the notable exception of the surface layer between 20°N and 30°N where concentrations approached a diffusion-limited regime, a region known to exhibit seasonal phosphorus limitation (*33*).

Among strains capable of transporting and assimilating all three, nitrogen was preferentially taken up in the order as follows: ammonia, nitrite, and nitrate. As a consequence of the high energy costs associated with nitrate and nitrite reduction, preference for reduced nitrogen was most pronounced in low-light environments, where a sensitivity analysis (Materials and Methods) indicated energy limitation, although there is also a small effect (in the same order) on encounter rates due to differences in molecular diffusivity. Several low light–adapted strains acclimated to growth in these low-light, high-inorganic nitrogen environments were further able to reduce the energy demands of nitrogen assimilation by redirecting a substantial portion (up to 48%) of the flux from the ATP-dependent glutamine synthetase–glutamate synthase complex (glutamate aminating) to the reduced form of nicotinamide adenine dinucleotide phosphate (NADP^+^)–dependent glutamate dehydrogenase (GDH; 2-oxoglutarate aminating). The auxiliary role of GDH in nitrogen assimilation under low-light, high-nitrogen conditions has been recognized in the model freshwater cyanobacterium *Synechocystis* (*34*) but not in *Prochlorococcus* (*35*).

Under resource-limiting conditions, growth rates can be further increased through the rebalancing of macromolecular pools to reduce the quota of that resource, thereby increasing yields. Particularly, in regions where inorganic nitrogen sources were scarce (e.g., in the upper water column of the subtropical gyres), protein content was reduced, leaving the remaining cellular mass to be partitioned predominantly between either carbohydrate or lipid stores. Because the energy requirements for de novo synthesis of lipids is considerably higher than of carbohydrates, allocating C to lipids was favored near the surface, while carbohydrates were relatively enriched just above the deep chlorophyll maximum depth interval. This solution points to lipid storage as an auxiliary electron sink to cope with high-light, low-nitrogen conditions. The resulting changes in the energy content of biomass can therefore be decoupled from synoptic changes in cellular C:N ratios, a prediction with implications for food web dynamics and trophic transfer efficiency (*36*). The PhysOpt algorithm also predicted subtle changes to other macromolecular pools, including reductions in P-containing compounds (found in RNA, in cell wall–associated lipopolysaccharides, and in other metabolites, vitamins, and cofactors) in the upper water column near 26°N where phosphate was most depleted (≤20 nM).

Changes in photophysiology and photosynthetic performance were predicted across latitudinal and vertical light gradients. In regions with high light and low nitrogen, the photoprotective pigment zeaxanthin increased while light-harvesting pigments α-carotene and divinylchlorophylls a and b decreased, providing a mechanism for nonphotochemical quenching of excess photons to be dissipated as heat. α-Carotene contributed a modest 22 ± 9% of total light-harvesting pigment absorption across all strains but appears to be somewhat favored by the HLII ecotype (28 ± 7%). These predictions are consistent with observations of *Prochlorococcus* isolates grown under variable light intensity and quality (*37*, *38*). Spectral tuning also influenced optimal pigmentation; the gradual shift in downwelling irradiance spectra favors the de novo synthesis of divinylchlorophyll b over divinychlorophyll a in deeper samples, a well-documented phenomenon in naturally occurring *Prochlorococcus* populations (*17*, *39*, *40*) and phytoplankton more broadly (fig. S14) (*41*).

**Fig. 3.**
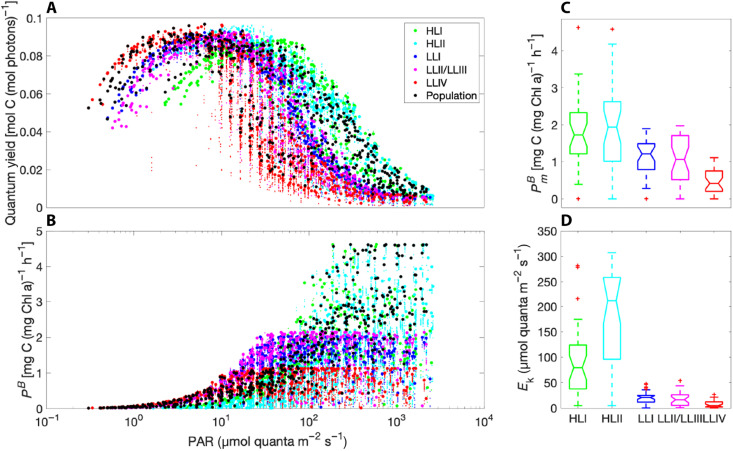
Photoacclimation and photosynthesis. (**A**) Predicted QY of photosynthesis (moles C fixed by ribulose 1,5-bisphosphate carboxylase per mole photons absorbed by light-harvesting pigments) over the range of in situ irradiance levels for all strains (small markers), ecotypes (large markers), and the total population (large black markers) across all samples. (**B**) Predicted photosynthetic rate normalized to the predicted chlorophyll against irradiance for all strains, ecotypes, and the total population across all samples. Box-and-whisker plots of meridional variations in (**C**) the assimilation number ($P_{m}^{B}$) and (**D**) the photosynthetic saturation irradiance (*E*_k_), derived from least-squares regression of predicted photosynthesis-versus-irradiance curves at each station.

Strain-specific differences in key photosynthesis parameters were derived from modeled photosynthesis versus irradiance curves at each station. The predicted meridional variation in the initial slope (α), assimilation number ($P_{\text{max}}^{B}$), and the photosynthetic saturation parameter (*E*_k_) was remarkably consistent with both in situ observations (*42*) and laboratory experiments (Fig. 3) (*17*). The quantum yield (QY) of photosynthesis, defined here as the moles of carbon fixed by ribulose bisphosphate carboxylase per mole of photons absorbed by the light-harvesting pigments, is a holistic measure of photophysiological states. In general, QY approached (but did not reach) a theoretical limit of 0.125 mol C (mol photons)^−1^ and declined because of the temperature dependence of biosynthetic rates at lower irradiances that covaried with in situ temperatures (Fig. 3). Subsurface maxima in the QY clustered primarily by ecotype, with overlapping optima from high to low light in the order HLII, HLI, LLI, LLII/III, and LLIV. The same general order was found for predicted optimal growth temperatures (OGTs) (data S4 and fig. S15), perhaps because of the covariation of temperature and light. Notably, this same order describes the phylogenetic evolution of the *Prochlorococcus* lineage, with deep-branching LLIV strains occupying the lower euphotic zone and more recently diverged ecotypes expanding their niche range toward the sea surface (*15*).

Several alternative electron flow pathways were active under high-light, low-nitrogen conditions, including the Mehler reaction, pseudo-cyclic electron flow around photosystem I, and ferredoxin-NADP^+^ reductase. In some strains, excess reductant is dissipated by the plastoquinone terminal oxidase (PTOX) reaction, while in other strains lacking PTOX, the generation of singlet oxygen and other free oxygen radicals is unavoidable, requiring glutathione or thioredoxin peroxidases. Partial recovery of the photorespiratory by-product 2-phosphoglycolate was only possible for a subset of the LLIV strains, while other ecotypes are known to excrete substantial amounts of the hydroxy acid glycolate (*43*). Although a comprehensive analysis of all strains across the transect was not possible, flux variability analysis (Materials and Methods) (*44*) indicated that glycolate excretion was favored by strains lacking a complete photorespiratory pathway in high-light, rather than low-light, conditions, supporting the notion of photorespiration as an additional alternative electron flow pathway (*45*, *46*).

### Metabolic microdiversity and the “nano-niche”

A survey of growth capabilities and pathway enrichment analysis among the core and accessory reaction subsets of the *Prochlorococcus* pangenome revealed the commonality of metabolic features at different taxonomic resolutions. The core subset, reactions common to all strains, included much of the photosynthetic, respiratory, and central carbon metabolism pathways, as well as the anabolic pathways for amino acids, lipids, light-harvesting pigments, vitamins, and cofactors. The accessory subset, reactions present in only some strains, was more widely distributed among pathways, especially those that enable access to nutrient sources in specific environments (fig. S15). For example, accessory genes associated with the LLIV clade included an enrichment in chitin-degrading and other polysaccharide-degrading enzymes and transporters, resources that are expected to supplement their energy-limited niche below the deep chlorophyll maximum depth. While our metabolic networks resolve the diversity of mixotrophic potential across the *Prochlorococcus* pangenome, comprehensive determinations of compound-specific dissolved organic matter concentrations in the marine environment remains an analytical challenge. Although exogenous organic substrate uptake was not included in our AMT simulations, ecotype-specific patterns were evident from an analysis of growth capabilities on various sole N, P, and S sources. For example, LLI and LLIV strains were universally able to grow on nitrite as a sole N source, while only four strains from other ecotypes shared that capability (fig. S16).

**Fig. 4.**
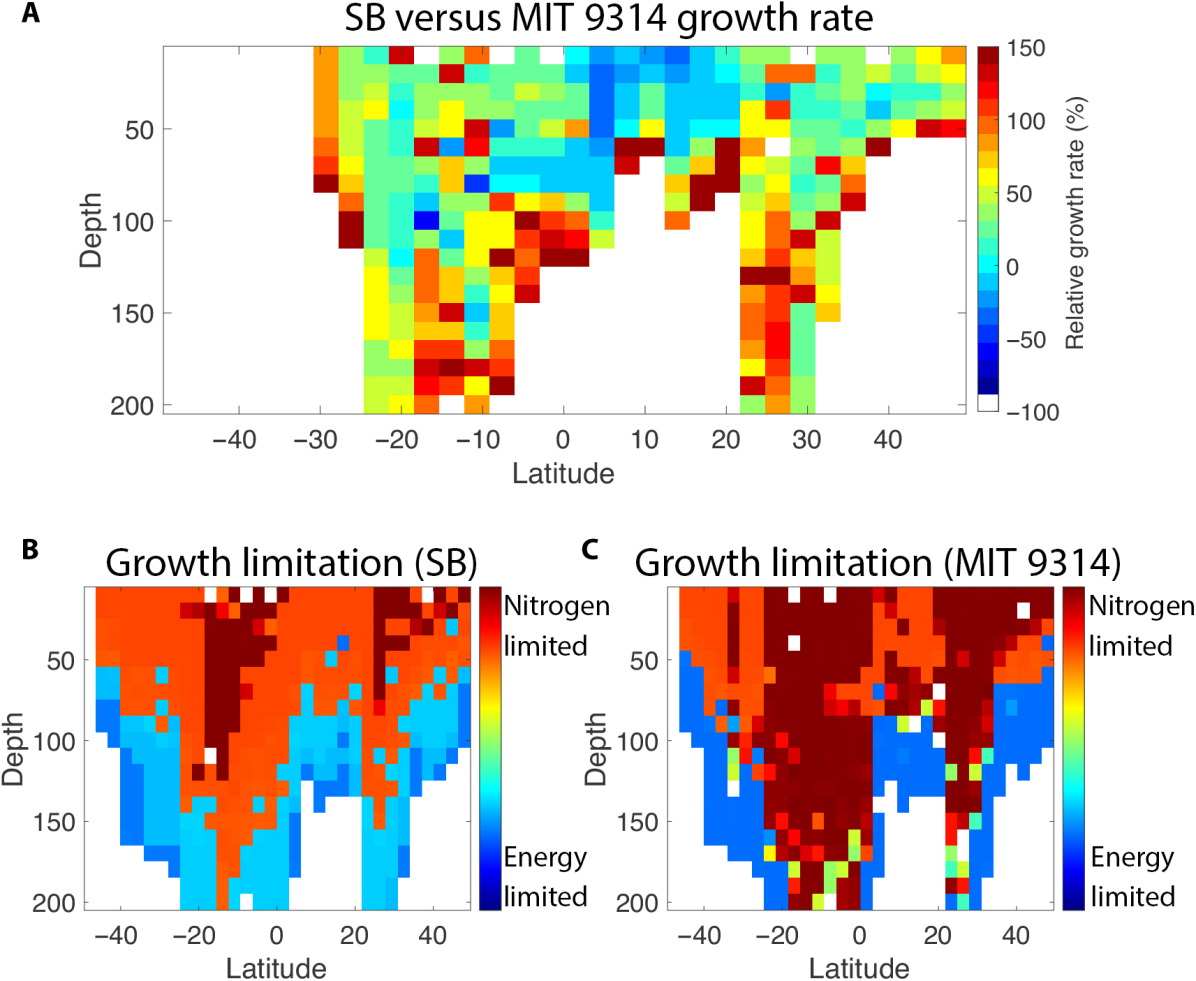
Metabolic microdiversity. (**A**) Meridional section of the relative growth rate of strains SB and MIT 9314. A positive value indicates the percentage increase in growth rate for strain SB relative to strain MIT 9314. (**B** and **C**) Meridional sections of resources limiting the growth of strains SB and MIT 9314. Warmer colors correspond to increasingly nitrogen-limited phenotypes, while cooler colors correspond to increasingly energy (light)–limited phenotypes.

While some metabolic strategies could be generalized at the ecotype level, even closely related strains with only subtle differences in their metabolic networks occupied different niches in physical and chemical space, coined a nano-niche (*47*). To illustrate this point, a head-to-head comparison was conducted between two HLII strains, MIT 9314 and SB, which are closely related both phylogenetically (*13*) and in terms of overlap in their metabolic networks (Jaccard similarity of 97.3%; fig. S2). Predicted growth rates of both strains were similar near the surface in the subtropics, but MIT 9314 outpaced SB in the surface layer of the equatorial region and SB outpaced MIT 9314 at higher latitudes and in deeper samples. A metabolite sensitivity analysis (Materials and Methods), used in this context as diagnostics of nutritional and energy status in key metabolites, indicated that the transition depth from nutrient limitation to light limitation occurred higher in the water column for SB, whereas MIT 9314 was more pervasively nitrogen limited (Fig. 4). Inspection of the subsets of reactions unique to each strain provided the mechanistic basis for their niche differentiation. MIT 9314 supplemented the tricarboxylic acid cycle intermediate oxaloacetate via the anapleurotic carbon fixation enzyme phosphoenolpyruvate carboxykinase (PEPCKase), increasing its relative fitness with respect to SB in the surface layer of the equatorial region where carbon was essentially growth limiting for this strain. The coupling of PEPCKase to a truly incomplete tricarboxylic acid cycle in *Prochlorococcus* (*48*) is potentially unique and has not been demonstrated in vivo. Near the deep chlorophyll maximum and at higher latitudes, SB leveraged its access to nitrate and nitrite transport and reduction to outpace MIT 9314, which relied on ammonia as its sole nitrogen source. The two strains converged in fitness below the 1% light depth because of the diminishing returns of oxidized nitrogen assimilation. Reactions attributed to the difference in niche optima between the two strains were validated with simulated knock-in mutants expressing the missing subsets in each. The resulting offset in the depth intervals for optimal growth were also reflected in their predicted OGTs (29.06°C for MIT 9314 and 27.86°C for SB; data S4).

Mechanistic, genome-scale modeling of cellular metabolism and physiology offers readily interpretable and, importantly, directly testable hypotheses in unprecedented detail. Moreover, models that resolve the molecular scale are needed to interface with the deluge of environmental omics data. However, the adoption of GEMs and constraint-based methods as prognostic tools in environmental microbiology and microbial ecology has been limited thus far (*49*–*51*). The traditional flux balance approach has routinely been applied to predict growth rates and yields of monoclonal isolates in chemostat, given a priori knowledge of not only internal stoichiometry and external resource availability but also, crucially, resource acquisition rates and biomass compositions, both of which vary widely in natural systems. Explicit, mechanistic modeling of acclimation processes (nutrient acquisition, photosynthetic architecture, and biochemical compositions) are therefore an important component of the model and a key factor in capturing the flexibility of relative fitness. By canvasing the microdiversity of physiological and metabolic traits within populations, our constraint-based optimization approach enables a bottom-up perspective on microbial population physiology, metabolism, and growth in diverse environments at multiple taxonomic resolutions. In addition to the many testable predictions of cell stoichiometry, nutrient uptake, metabolic rates, and photophysiology, the correspondence of the predicted optimal growth rate patterns with observed abundances is intriguing, and the emergent relationship could provide a calibration for a density-dependent mortality closure with broader application.

Implementation of this framework in regions beyond the AMT transect, where appropriate environmental data are already available, would provide testable predictions on the relative abundance, composition, and activity of *Prochlorococcus* ecotypes. However, because of the streamlined nature of *Prochlorococcus*, much of the metabolic diversity within the accessory subset of the pangenome is associated with the transport and metabolism of exogenous organic substrates that are neither routinely measured in situ nor resolved in our current implementation. Exchange of these resources within *Prochlorococcus* populations may provide stability to strain diversity through cross-feeding and division of labor. This is one avenue in which pangenome-scale modeling may contribute in the future, because many specific metabolite exchanges could, in theory, be resolved. Allowing the virtual organisms to feed back on their resource environment could lead to prognostic applications (*52*), perhaps embedded in a coarser-grained general population. Although currently still computationally prohibitive, with effort on code development and optimization, this is a plausible goal. The degree of metabolic detail resolved makes these tools a good prospect for exploring community interactions.

## MATERIALS AND METHODS

### Environmental data

In situ and remote sensing data were used as inputs for simulations. A suite of 66 measured in situ variables from the 2003 AMT-13 cruise, including temperature, nutrients, cell abundances, and optical properties, are described in detail in the Supplementary Materials (section S1.1) and are provided as a gridded dataset (data S2). To compute downwelling irradiance, we combined in situ optical profiler diffuse spectral attenuation coefficients with synoptic measurements of satellite cloud cover as a mask over a model of clear-sky direct and diffuse spectral radiation. Simulations conducted in this study were restricted to a subset of 32 stations (from a total of 78 casts at 54 stations) for which ecotype abundance data (*9*) were available.

### Biochemical compositions

Biochemical compositions, physiological traits, and constraints on their ranges were assigned to each strain. Initial compositions represent the nutrient-replete batch-acclimated state of isolates as a starting point for acclimation. Data assigned to each strain were derived both from literature sources and from measurements taken for this study. Metabolomic data were collected for five *Prochlorococcus* strains under optimal batch conditions: MED4 (HLI), MIT 9312 (HLII), NATL2A (LLI), MIT 1304 (LLII/III), and MIT 9313 (LLIV). Strain MED4 was analyzed for macromolecular compositions (carbohydrate, lipid, protein, DNA, RNA, and divinylchlorophyll a) as well as elemental composition (particulate C, N, and P) and dry weight as in (*53*) (with modifications) under nutrient-replete and nitrogen and phosphorus stress conditions. Detailed analytical methods and a summary of literature datasets used in this study are provided in the Supplementary Materials (section S1.5 and data S7).

### Pangenome-scale metabolic network assembly

An aggregate GEM was reconstructed to represent the full set of metabolic functions encoded in the pangenome of *Prochlorococcus* (PanGEM). The current pangenome consists of 77 sequenced isolates, seven metagenome-assembled genomes, and 564 single-cell amplified genomes, comprising 866,894 unique open reading frames. Translated coding sequences were mapped to KEGG orthologs (KOs) using a bidirectional blast with KEGG’s kofamKOALA, pairing the number of unique KOs to 2084. A database containing nucleotide sequences, translated amino acid sequences, header strings, database identifiers (National Center for Biotechnology Information, Joint Genome Institute, and KEGG), metadata, and descriptive statistics for each genome was compiled; this database was the basis for draft reconstruction of the PanGEM network. The draft reconstruction was then manually curated following a standard process (section S1.3) (*54*).

### Semiautomatic strain GEM reconstruction

A novel approach to automatically extract functional, stoichiometrically consistent strain GEMs from the PanGEM was developed. By definition, any strain is a subset of the PanGEM; however, simply excluding the remaining reactions often results in lethal deletions. In lieu of manual curation for each strain, we use a CS algorithm to identify an essential subset of reactions that are required to synthesize all biomass components de novo and satisfy the growth objective. From this essential reaction subset, we append reactions specific to each strain. A detailed description of PanGEM and the CS algorithm is provided in the Supplementary Materials (section S1.4).

### Flux balance analysis

A chemical flux balance can be derived from the law of mass action (section S1.2). Assuming a steady state, a set of optimal flux distributions can be predicted that maximizes an objective function by solving the linear program

$$\begin{array}{cc} \overset{\text{maximize}}{v\in {\mathbb{R}}^{n}} & c^{T}v\\ \text{subject to} & \begin{array}{l} \mathrm{Sv}=0,\\ v^{\mathrm{lb}}\leq v\leq v^{\mathrm{ub}} \end{array} \end{array}$$ (1)

where **S** is the stoichiometric matrix, **v** is the vector of fluxes and its respective lower and upper bounds (**v^lb^** and **v^ub^**), and **c** is a predefined coefficient vector whose entries define the weighted set of reactions that determine fitness. Here, the objective function is defined as growth rate, the rate of synthesis of 1 g of ash-free dry biomass of specified composition. Several variations of FBA and sensitivity analyses are used throughout this article, as described in the Supplementary Materials (section S1.2).

### Microbial Simulation Environment

Two sequential acclimation steps, OptTrans and PhysOpt, are performed on each strain GEM at each environmental grid point. Physiological data specific to each strain, or to its closest phylogenetic relative, are initially assigned to the corresponding GEM to represent its phenotype when grown under optimal laboratory conditions. Physiological data, collected as a part of this study (data S4 to S7) and from the literature (data S8), include cell size spectra, biochemical compositions (macromolecular pools, pigments, and metabolites), maximum carbon fixation rates, and maintenance requirements. Boundary constraints for all parameters (e.g., molecular compositions, cell size, and metabolic rates) are assigned to each strain GEM by compiling experimental data collected under a wide range of growth conditions. The first bilevel optimization, OptTrans, searches for the optimal distribution of metabolic fluxes, transporter abundances, and cell size that maximizes fitness, given the external concentration of each nutrient (ammonia, nitrite, nitrate, and phosphate). Quantitative proteomics data and molecular modeling are used to derive catalytic rates and transmembrane domain dimensions for each transporter, necessary to predict external substrate concentration–dependent uptake rates (*32*). The calculated optimal uptake rates, cell sizes, and transporter abundances are then used to constrain the second bilevel optimization, PhysOpt. Given our experimentally determined constraints on cellular biochemical compositions (data S5), PhysOpt searches for the optimal biomass composition of several macromolecular pools (e.g., protein, DNA, RNA, cell wall, lipid, carbohydrates, minerals, vitamins, osmolytes, and other metabolites). Simultaneously, pigment compositions (the light-harvesting pigments divinylchlorophylls a and b, α-carotene, and the photoprotective pigment zeaxanthin) are optimized according to the alignment of pigment-specific absorption spectra with the observed in situ downwelling irradiance spectrum, again with the objective of maximizing fitness. This coupling therefore takes into account the costs of synthesizing each macromolecular pool or pigment de novo, in the holistic context of total cellular nutritional status, photon flux demands, metabolic capabilities, and biomass composition, specific to each strain. The entire MSE procedure is intended to mechanistically represent the processes by which microbes acclimate to their environment at the molecular scale, while being faithful to the trade-offs of altering their physiology. A more thorough discussion and accompanying illustrations for OptTrans, PhysOpt, and the transport and photosynthesis models are provided in the Supplementary Materials (section S1.6).

### Temperature dependence of metabolic rates

Growth rates and metabolic fluxes were adjusted for temperature effects by comparing in situ growth temperatures to a model of temperature-dependent growth, parameterized for each strain. Our approach provides a strain-specific parameterization while avoiding the effects of adaptive laboratory evolution, which can be substantial (*55*). First, the Arrhenius activation energy *E*_a_ was calculated on the basis of growth rates determined in batch cultures of 12 *Prochlorococcus* strains (*9*, *56*). A modification of a previously proposed model (*57*), but which preserves the Arrhenius parameterization, was used to capture the dynamics of a dimensionless growth rate constant that was then applied to metabolic rates and growth rates. The OGT parameter was predicted for each strain from proteome sequences using the machine learning algorithm TOME-cool. Among several algorithms, a support vector machine regression achieved the best performance (coefficient of determination score, *R*^2^ = 0.88). The OGT dataset from TOME 1.0 (*26*) was expanded to include additional psychrophilic and psychrotolerant taxa (*58*, *59*), resulting in a training dataset of 6020 microorganisms (https://github.com/EngqvistLab/tome_cool; section S1.7).

## Supplementary Materials

### This PDF file includes:

Supplementary Materials and Methods Tables S1 and S2 Figs. S1 to S19 Legends for data S1 to S6 References
