# Supplementary material for: Basin-scale biogeography of marine phytoplankton reflects cellular-scale optimization of metabolism and physiology
Source: Sci Adv. 2022 Jan 21;8(3):eabl4930. doi: 10.1126/sciadv.abl4930 (PMC8782455; doi:10.1126/sciadv.abl4930)
Supplement: Supplementary file 1 — Supplementary Materials and Methods Tables S1 and S2 Figs. S1 to S19 Legends for data S1 to S6 References [file sciadv.abl4930_sm.pdf]

Science Advances
AAAS

Supplementary Materials for

Basin-scale biogeography of marine phytoplankton reflects cellular-scale optimization of metabolism and physiology

John R. Casey*, Rene M. Boiteau, Martin K. M. Engqvist, Zoe V. Finkel, Gang Li, Justin Liefer, Christian L. Müller, Nathalie Muñoz, Michael J. Follows

*Corresponding author. Email: jrcasey@mit.edu

Published 21 January 2022, *Sci. Adv.* **8**, eabl4930 (2022)
DOI: 10.1126/sciadv.abl4930

The PDF file includes:

Supplementary Materials and Methods
Tables S1 and S2
Figs. S1 to S19
Legends for data S1 to S6
References

Other Supplementary Material for this manuscript includes the following:

Data S1 to S6

1 Methods

1.1 Environmental data

Data were compiled from the Atlantic Meridional Transect (AMT-13) cruise of the RSS James Clark Ross in the fall of 2003 from the English Channel to the Falkland Islands (Islas Malvinas; fig. 2a), provided by request from the British Oceanographic Data Centre (BODC). 66 measured variables were compiled (data S2), including hydrographic data, spectral light data, nutrient concentrations, pigment concentrations, particulate and dissolved elemental compositions, microbial community compositions, and biological rate measurements. Most measurements were taken from bottle samples collected in depth profiles at 10 m resolution in the upper 100 m, and 20 m to 50 m resolution to 300 m, and additional samples were sometimes collected at the deep chlorophyll maximum depth. *Prochlorococcus* ecotype abundances, determined by qPCR, were included for 32 stations as reported in [9]. Simulations conducted in this study were restricted to this subset of 32 stations (from a total of 78 casts at 54 stations) for which synoptic ecotype abundance data were available, although we provide the complete transect dataset with permission from the BODC (data S2).

CTD Vertical profiles of pressure, temperature, salinity, dissolved oxygen, and chlorophyll a were collected using a Sea-Bird SBE 911plus CTD. Data were processed at 1 m resolution and calibrated with *in situ* samples. Potential density was calculated using the UNESCO 1983 algorithm. The deep chlorophyll maximum depth interval was defined as $\pm$10m from the maximal CTD fluorescence depth.

Spectral irradiance Spectral downwelling irradiance profiles were conducted at 22 stations along the transect, using a free-falling optical profiler equipped with 8 wavebands in the photosynthetically active radiation range (412 nm to 685 nm). Diffuse spectral attenuation coefficients were measured at 1 m intervals at each station. To capture light dynamics on the photoacclimation timescale, an 8-day average cloud optical thickness τ_c was applied as a mask to a model of direct and diffuse clear-sky spectral radiation at each station. Level 2 Aqua cloud products were retrieved from NASA's Atmosphere Archive and Distribution System (https://earthdata.nasa.gov/eosdis/daacs/laads) at 0.1 degree resolution for an area of 3 x 3 pixels centered at each station at the time of sampling. A spatial average was calculated for each station, omitting pixels with missing data. Clear-sky direct

and diffuse spectral radiation was calculated at 2 nm resolution using the SMARTS algorithm (Simple Model of the Atmospheric Radiatived Transfer of Sunshine, National Renewable Energy Laboratory; https://www.nrel.gov/grid/solar-resource/smarts.html) at local noon for the spectral attenuation measurement band. Spectral downwelling irradiance (E_d;W m^{-2} nm^{-1}) was then calculated for each station at discrete depths as

$$E_d(\lambda, z) = \int_{\lambda_{min}}^{\lambda_{max}} E_d(\lambda, 0) e^{-k_d(z)z} d\lambda \tag{2}$$

where λ is the wavelength (nm), and k_d is the attenuation coefficient (m^{-1}) measured at each depth z (m). To match the resolution of both the surface irradiance and the pigment spectral absorption measurements (described in sections 1.5 and 1.6), a spline interpolation was applied in the wavelength domain of k_d at 2 nm resolution.

Nutrients Nutrients used in simulations included ammonium, nitrite, nitrate, and phosphate. High- and low-sensitivity methods were implemented for their appropriate concentration intervals on each cast, and where overlapping data were available the high-sensitivity measurement was chosen. Nitrate concentrations were calculated as the difference between spectrophotometrically determined nitrite and nitrate+nitrite. Silicate and the reduced sulfur compounds dimethylsulfoxide (DMS) and dimethylsulfionopropionate (DMSP) were quantified on a subset of stations, and are included in the dataset but were not used in simulations. Values below the detection limit were encountered in 4 stations (data S2), and in those cases concentrations were assigned to the detection limit.

Particulate carbon, nitrogen, and chlorophyll Particulate organic carbon and nitrogen were determined by filtration onto glass fiber filters (Whatman GF/F), acidification to aspirate inorganic carbon, and elemental analysis.

Size fractionated chlorophyll a (including divinyl chlorphyll a) concentrations were measured fluorometrically in the intervals 0.2-5 μm, 5-10 μm, and >10 μm using polycarbonate membrane filters, as well as on glass fiber filters (Whatman GF/F). An additional 14 pigments were quantified using HPLC methods, also extracted from GF/F's.

Flow cytometry *Prochlorococcus*, *Synechococcus*, Cryptophytes, Coccolithophores, heterotrophic bacteria, picophytoplankton, and nanophytoplankton population abundances were determined by flow cytometry, after fixation with 1% paraformaldehyde and staining with SYBR Green I (Molecular Probes) using a cuvette-based cell sorter (FACSort, Becton Dickinson).

Bulk rate measurements Bulk rate measurements included gross primary production (GPP), net primary production (NPP), community respiration (CR), ^{14}C-primary production (^{14}C-PP), and nitrogen uptake rates. Deckboard incubations at *in situ* temperatures and light levels were used for all rate measurements. GPP, NPP, and CR rates were determined by 24 hour *in vivo* oxygen changes, following the Winkler titration method [85]. ^{14}C-PP incubations were approximately 12 hours, from dawn to dusk, and were processed following [60]. At most stations, subsets from the surface and 1% light depth were size fractionated at 0.2-2 μm and >2 μm. Nitrate, ammonium and urea uptake rates were determined using ^{13}C and ^{15}N tracers spiked in approximately 10 hour deckboard incubations, again at *in situ* temperatures and light levels, and dark bottles were also included.

Further details of collection methods, analysis and data processing of samples collected on the AMT-13 cruise can be found at `https://www.bodc.ac.uk/projects/data_management/uk/amt/`.

1.2 Flux balance analysis

A chemical flux balance can be derived from the Law of Mass Action. A system of n chemical reactions involving m chemical species at concentrations x_i can be expressed in terms of the stoichiometry of their reactants a_{ij} and products b_{ij},

$$a_{1j}[x_1] + a_{2j}[x_2] + \ldots + a_{mj}[x_m] \underset{k_j^-}{\overset{k_j^+}{\rightleftharpoons}} b_{1j}[x_1] + b_{2j}[x_2] + \ldots + b_{mj}[x_m]. \quad (3)$$

The jth reaction proceeds in the forward and reverse directions according to the rate constants k_j^+ and k_j^-, respectively. Thus, the net rate of change in the concentration of the ith chemical species is given by the sum:

$$\frac{dx_i}{dt} = \sum_{j=1}^{n}(b_{ij} - a_{ij})(k_j^+[x_1]^{a_{1j}}[x_2]^{a_{2j}}\ldots[x_m]^{a_{mj}} - k_j^-[x_1]^{b_{1j}}[x_2]^{b_{2j}}\ldots[x_m]^{b_{mj}}). \quad (4)$$

The system of reactions can be represented by an $m \times n$ matrix of the directed stoichiometric coefficients $S_{ij} = b_{ij} - a_{ij}$, so that reactants and products are assigned negative and positive values, respectively. The forward and backward rates of each reaction, described by the second term of the sum, can also be represented by a vector of fluxes v_j, such that

$$\frac{dx_i}{dt} = \sum_{j=1}^{n} S_{ij}v_j. \quad (5)$$

It is often the case that both x and k are unknown, so the system of reactions is stoichiometrically balanced but the rates are effectively unbounded. The steady-state scenario ($Sv = 0$) is often imposed in flux balance analysis (FBA) as a simplifying assumption which implies that, over some time interval Δt, there is no net change in the concentration of any internal chemical species. A relaxation of this assumption in non-steady-state situations can be found elsewhere [61], but ideally one should limit their interpretation of FBA predictions to the chemostat.

Wherever possible, knowledge of the reaction kinetics and thermodynamic parameters are leveraged to establish directionality, and in some cases, capacity constraints (i.e., v_{max}; discussed below). Reactions which are reversible remain unbounded, while reactions which are expected to only proceed in the forward or backward direction are constrained by lower bounds v_j^{lb} or upper

bounds v_j^{ub} of zero, respectively. It is critical that all reactions included in the network be balanced for mass and charge, and that each chemical species be stoichiometrically constistent. In practice, however, a number of extracellular chemical species are unconstrained by mass balance and are allowed to freely exchange with the system boundary, enabling flux through the network and the net synthesis of biomass.

Assuming a steady-state, a set of optimal flux distributions can be predicted that maximizes "fitness" by solving the linear program

$$\begin{aligned} \underset{v \in \mathbb{R}^n}{\text{maximize}} \quad & c^T v \\ \text{subject to} \quad & Sv = 0, \\ & v^{lb} \leq v \leq v^{ub}, \end{aligned} \tag{6}$$

where c is a predefined coefficient vector whose entries define the weighted set of reactions that determine fitness. Here, fitness is defined as growth rate, the rate of synthesis of one gram of ash-free dry biomass of specified composition.

Although FBA will find a global maximum function value, the stoichiometric matrix S is typically underdetermined. The flux vector v is not unique, and S often contains thermodynamically infeasible loops. One strategy to retrieve a more sparse solution and to eliminate loops is to minimize the l_1-norm of the flux vector [62]. This is achieved with a second linear program. Assume that the previous program results in fitness $f = c^T v$. To retrieve a sparse and unique solution, we minimize the l_1-norm of the flux vector v subject to the additional linear constraint $f = c^T v$.

$$\begin{aligned} \underset{v \in \mathbb{R}^n}{\text{minimize}} \quad & ||v||_1 \\ \text{subject to} \quad & c^T v = f, \\ & Sv = 0, \\ & v^{lb} \leq v \leq v^{ub} \end{aligned} \tag{7}$$

When the linear constraint prohibits a sparse solution of v, one can relax the constraint within a predefined tolerance $\delta \in [0, 1]$ and solve the above problem using $c^T v \geq \delta f$ instead. To further explore alternative optima of the FBA solutions, flux variability analysis (FVA) is implemented to identify the maximal and minimal fluxes of each reaction while sustaining the optimal function value within a predefined tolerance. In FVA, the extrema are determined in two sequential optimizations (maximizing and minimizing) for each reaction v_j, holding the objective value $c^T v = f$ from Eq. (7) within tolerance δ. For brevity, we show only the minimization step:

$$
\begin{aligned}
\underset{v_j}{\text{minimize}} \quad & v_j \\
\text{subject to} \quad & Sv = 0, \\
& c^T v \geq \delta f, \\
& v^{lb} \leq v \leq v^{ub}
\end{aligned} \tag{8}
$$

A measure of the sensitivity of the objective function value to each metabolite is given by the dual solution to the FBA linear program (Eq. 5). Dual variables (of length n) λ_1 and λ_2 are assigned to the lower and upper bounds on each reaction. In our application, the biomass formation reaction v_B is maximized, so the sensitivity of growth rate to the supply of each metabolite ($\gamma_i = \frac{dv_B}{dx_i}$) is given by the minimization

$$
\begin{aligned}
\underset{\lambda_1 \in \mathbb{R}^n, \lambda_2 \in \mathbb{R}^n}{\text{minimize}} \quad & \lambda_1 v^{lb} + \lambda_2 v^{ub} \\
\text{subject to} \quad & c^T = \gamma^T S + \lambda_2^T + \lambda_1^T, \\
& \lambda_1 \leq 0, \lambda_2 \geq 0
\end{aligned} \tag{9}
$$

Critically, the above optimizations require the assignment of appropriate metabolic rate constraints and biochemical compositions for each strain in each environment. In the following sections, we describe the process of reconstructing the metabolic networks (sections 1.3 and 1.4), specifying strain-specific constraints on biochemical compositions and fluxes (section 1.5), incorporating physiological acclimation processes (section 1.6), and accounting for temperature effects (section 1.7).

1.3 Pangenome-scale metabolic network assembly

An aggregate GEM was reconstructed to represent the full set of metabolic functions encoded in the pangenome of *Prochlorococcus* (PanGEM). The current pangenome consists of 77 sequenced isolates, 7 metagenome assembled genomes, and 564 single cell amplified genomes, comprising 866,894 unique ORFs. Gene assemblies were retrieved from the JGI Integrated Microbial Genomes and Microbiomes portal (IMG; http://img.jgi.doe.gov). Translated coding sequences were mapped to KEGG orthologs (KOs) using a bi-directional blast with KEGG's kofamKOALA, paring the number of unique KOs to 2084. A database containing nucleotide sequences, translated amino acid sequences, header strings, database identifiers (NCBI, IMG, and KEGG), metadata and statistics for each genome was compiled; this database was the basis for draft reconstruction of the PanGEM network.

Gene-protein-reaction (GPR) associations were established for all functionally annotated metabolic KO's using the KEGG API. Reactions linked to each KO were parsed into their participating metabolites and stoichiometric coefficients, tabulated and annotated for the draft reconstruction. Wherever possible, each reaction and metabolite was associated with unique identifiers from KEGG, MetaNetX, NCBI PubChem, EMBL-EBI ChEBI, EMBL-EBI RHEA, Enzyme Commission numbers, and InChI. In rare cases, multiple identifiers were linked to a single entry.

Each metabolite linked to a GPR was assigned a chemical formula, charge, and molecular weight using KEGG and PubChem databases. In entries where the molecular formula was generic (i.e., wherever an R-group was present), molecular weights and charges were recorded only for the parent moiety. For polysaccharides, the number of monomers was assigned based on glycogen containing 10-mers, solely for convenience. Each lipid head group and their associated acyl chain lengths and saturation states were assigned based on data available for the HLI strain MED4 [63]. Standard Gibbs formation energies ($\Delta_f G^{'o}$) for each metabolite were predicted using the eQuilibrator tool [64] at pH = 7.4, ionic strength of 0.7 M (appropriate for marine microbes), and an intracellular concentration of 1 mM, however intracellular concentrations were unknown and may introduce error to *in vivo* $\Delta_f G^{'o}$ predictions. Entries with effectively zero-mass (e.g., photons, excitons, heat) were assigned a charge and their own 'element' e.

In addition to internal metabolic reactions with KO associations, several other sets of reactions were added to the model including: (1.) transport, exchange, and spontaneous reactions, (2.) light-harvesting and photosynthetic reactions, (3.) biomass formation reactions, and (4.) 'gap-filled' reactions with no known GPR, described in more detail below.

1. KOs were manually assigned to transport reactions using the TransportDB database `http://www.membranetransport.org/`, but the direction of transport was not always known. For example, in the case of ATP binding cassette transporters, whether the membrane receptor domain is inward facing (exporter) or outward facing (importer) was not assumed and was instead parsed into two irreversible reactions. In the case of symporters and antiporters, transporter flux bounds were assumed to be reversible. Exchange reactions were added for all external metabolites, including 'biomass', and were allowed unbounded flux with unconstrained 'boundary' metabolites.

2. Photons are absorbed by each pigment separately, according to the *in situ* downwelling irradiance spectrum and the pigment-specific absorption spectrum (Section 1.6). Non-photochemical quenching was also explicitly represented for the relaxation of each pigment in the form of heat, which was included as a 'metabolite.' Reactions involving the photoprotective pigment zeaxanthin were included separately for its co-localized quenching of singlet, doublet, and triplet excited divinyl chlorophylls *a* and *b*. The role of the secondary light-harvesting pigment α-Carotene in *Prochlorococcus* photosystems remains unclear; however, we currently consider it to be a light-harvesting pigment and allow exciton transfer by the relaxation of a singly-excited electronic intermediate state of α-Carotene to the ground state, transferring an exciton to either photosystem I or II:

$$\begin{aligned} \alpha Carotene + Photon_{\alpha Carotene} &=> \alpha Carotene^{1*} \\ \alpha Carotene^{1*} &=> \alpha Carotene + Exciton \end{aligned}$$

In the cases of divinylchlorophyll's a and b, the series of singly-, doubly-, and triply-excited electronic states are explicitly represented both for their excitation and relaxation intermediate reactions:

$$\begin{aligned} DVChla/b + Photon_{DVChla/b} &=> DVChla/b^{1*} \\ DVChla/b^{1*} + Photon_{DVChla/b} &=> DVChla/b^{2*} \\ DVChla/b^{1*} &=> DVChla/b + Exciton \\ DVChla/b^{2*} + Photon_{DVChla/b} &=> DVChla/b^{3*} \\ DVChla/b^{2*} &=> DVChla/b^{1*} + Exciton \\ DVChla/b^{3*} + Oxygen(th) &=> Oxygen^{-}(th) + DVChla/b \end{aligned}$$

Although the mechanism of the photoprotective pigment zeaxanthin is not known in *Prochlorococcus*, there is evidence [65] in *Synechococcus* that it is bound to the light harvesting complex as accessory antennae, suggesting that it may directly strip photons from its neighboring chlorophylls through resonance energy transfer, and further provides a mechanism to quench singlet oxygen in the thylakoid lumen (th). The relaxation of singly-excited zeaxanthin dissipates to heat (non-photochemical quenching), thus we chose to represent its function as:

$$\begin{aligned}
Photon_{DVChla/b} &=> Photon_{Zeax} \\
Zeaxanthin + Photon_{Zeax} &=> Zeaxanthin^{1*} \\
Zeaxanthin + Oxygen^{-}(th) &=> Zeaxanthin^{1*} + Oxygen(th) \\
Zeaxanthin^{1*} &=> Zeaxanthin + Heat
\end{aligned}$$

3. Biomass formation reactions were partitioned into individual macromolecular synthesis reactions and a lumped biomass reaction composed of the fractional weights of each macromolecule. The macromolecular pools include:

   (a) Protein - containing 21 amino acids (mmol g protein^{-1}) and requiring a fixed polymerization cost of 32 mmol ATP g protein^{-1}:

   (b) Lipid - containing monogalactosyldiacylglycerol (C16:0), digalactosyldiacylglycerol (C16:1, C18:0), sulfoquinovosyldiacylglycerol (C16:1, C18:0, C18:2), and phosphatidylglycerol (C18:1, C17:1).

   (c) Carbohydrate - containing only glycogen

   (d) DNA - containing each deoxyribonucleotide in the proportions of each full genome sequence.

   (e) RNA - containing each ribonucleotide in the proportions of the full genome sequence, transcribed.

   (f) Cell Wall - containing crosslinked peptidoglycans, UDP-diphosphate, and lipid A disaccharide.

   (g) Ions and Metals - containing the major ions of potassium, calcium, sodium, chloride, and magnesium, as well as the trace elements cadmium, cobalt, copper, iron (II), a guanylyl cofactor bound molybdenum, strontium and zinc.

   (h) Other pigments - containing a subset of pigments which may be synthesized by particular strains, but were not considered major

light harvesting or photoprotective pigments. These included phycocyanobilin, phycoerythrobilin, 7'8-dihydro-β-carotene, trans-ζ-carotene, β-carotene, δ-carotene, γ-carotene, and lycopene.

(i) Nucleosides and nucleobases - containing a set of 19 nucleosides, deoxynucleosides and their bases.

(j) Vitamins and cofactors - containing the vitamins biotin, pseudocobalamin, riboflavin, and thiamin diphosphate, as well as the dinucleotide cofactors NAD(P)H, THF, 5-THF, 5'10-MeTHF, FAD, CoA, acetyl-CoA, and malonyl-CoA.

(k) "BioPool" - containing a subset of metabolites which are known to be present at sufficiently high concentrations in cells but do not belong to any of the other macromolecular pools. These include chorismate, glutathione, heme, S-adenosyl-L-methionine, and spermidine. Metabolites identified in our metabolomics data are appended to this subset.

(l) Biomass - this is the lumped reaction on a weight basis (g of macromolecule g biomass^{-1} and includes each of the previously described macromolecular pools in their respective proportions and a growth associated maintenance ATP requirement which is cell size dependent (section 1.5).

4. Reactions with no gene or protein association, but which were required for biomass synthesis on minimal medium were added. This 'gap-fill' subset has no candidate GPR association and therefore requires considerable manual inspection and literature review to rationalize the choice of including. In most cases, the choice was simple, where a single missing reaction in a linear pathway could be added; however, in some rare cases the decision was more nuanced. We therefore have low confidence in their presence and have flagged these reactions to encourage future functional genomics insights and improvement to the quality of the model. The number of gap filled reactions was 36 (2.4% of all reactions).

The process from draft to final reconstruction followed procedures detailed previously [54, 66] and fall outside the scope of this article. Ensuring stoichiometric consistency, identifying unbalanced reactions and metabolites, detecting dead-ends, leaks and orphaned reactions, as well as a multitude of other tasks were conducted using the Matlab toolboxes COBRA (version 2.21.1; [54]) and RAVEN (version 2.4.0; [67]), both of which are extensively documented.

1.4 Semi-automatic strain GEM reconstruction

A novel approach to automatically extract functional, stoichiometrically consistent strain GEMs from the PanGEM was developed. By definition, any strain is a subset of the PanGEM, however simply excluding the remaining reactions often results in lethal deletions. In lieu of manual curation for each strain, we employ a strategy to identify an essential subset of reactions which are required to synthesize all biomass components *de novo* and satisfy the growth objective.

While a minimal essential reaction subset is possible, flexibility and redundancy in metabolic networks introduces some ambiguity in how we might define 'essential.' The removal of a key reaction may divert resources through a less efficient pathway, or one which cannot stoichiometrically carry the flux required to achieve maximal growth rates. Instead of searching for a minimal essential reaction subset, we identify an essential reaction subset which still allows for growth at some proportion $\epsilon \in [0, 1]$ of the maximal growth rate f. To do this, we implement a compressed sensing linear program which iteratively solves a constrained l_1-norm minimization of fluxes over a range of function values ϵf. The sparsest set of fluxes which satisfies the threshold $c^T v \geq \epsilon f$ is taken to be the essential reaction set $\mathfrak{E}$. The choice of a suitable ϵ value depends on the application, however in our case the most conservative approach was taken ($\epsilon = 1 - tol$, where tol is the interior point solver step tolerance). One can interpret this choice as taking the minimal set of reactions required to satisfy maximal biomass synthesis rates. To populate the complete metabolic network for each particular strain S, we append GPR associations unique to a strain's genome $\mathfrak{V}$, such that $S = \mathfrak{E} \cup \mathfrak{V}$.

$$
\begin{aligned}
\underset{v \in \mathbb{R}^{2n}}{\text{minimize}} \quad & ||v||_1 \\
\text{subject to} \quad & c^T v \geq \epsilon f, \\
& v^{lb} \leq v \leq v^{ub}, \\
& S^{+} v \geq 0, \\
& S^{-} v \leq 0
\end{aligned}
\tag{10}
$$

A possible shortcoming of our approach is that the appended set $\mathfrak{V}$ is assumed to be complete but may contain gaps. This decision reflects our limited knowledge of strain-specific growth capabilities. Coupling our approach to an automated gap filling method (e.g., ReFill; [68]) may provide additional metabolic capabilities, albeit at the risk of spurious additions. We note that, even without this step, predicted metabolic capabilities (fig S16) for the subset of strains that we do have experimental evidence for were

comprehensively captured by our algorithm. The table below summarizes a pairwise comparison of experimental and model predicted growth capabilities of nine strains on six sole nitrogen and phosphorus sources:

	Model	Experimental
Growth	22	23
No Growth	8	7

The PanGEM reconstruction steps and the CS algorithm are provided as a Matlab toolbox which can be accessed in a GitHub repository `https://www.github.com/jrcasey/PanGEM`. Strain model reconstruction was applied only to isolates with complete genomes. Metagenome assembled genomes and single cell assembled genomes were typically incomplete, resulting in large gaps in metabolic networks which would require other approaches.

1.5 Biochemical and physiological customization of strain GEMs

Biochemical compositions, physiological traits, and constraints on their ranges were assigned to each strain. Initial compositions represent the nutrient-replete batch acclimated state of isolates, as a starting point for acclimation. Data assigned to each strain were derived both from literature sources and from measurements taken for this study.

GAM and NGAM Growth associated maintenance (GAM; fmol ATP cell^{-1} h^{-1}) and non-growth associated maintenance (NGAM; fmol ATP cell^{-1}) ATP requirements were calculated based on allometric relationships [69]:

$$GAM = 44.7V^{0.97} \tag{11}$$

$$NGAM = 0.65V^{0.88} \tag{12}$$

where V is the cell volume (μm^3). NGAM costs were included as fixed upper and lower bounds on an ATP hydrolysis drain reaction. To differentiate other GAM ATP costs from structural biosynthesis ATP costs of macromolecular pools, the polymerization costs of amino acids, nucleic acids, lipids, and lipopolysaccharides were separately assigned to each respective macromolecule synthesis equation from the total predicted GAM value. Residual GAM costs were included in the bulk biomass equation.

Metabolomics Metabolomic data were collected for five *Prochlorococcus* strains: MED4 (HLI), MIT 9312 (HLII), NATL2A (LLI), MIT 1304 (LLII/III), and MIT 9313 (LLIV). Cell pellets were resuspended in 400 μL of water and lysed by bead beating with 0.1 mm diameter zirconia beads at 4°C for 3 min. Solutions were clarified by centrifugation at 2000 rpm for 10 min at 4°C. Total protein content of lysates was measured by the Pierce bicinchoninic acid (BCA) Protein assay (Thermo Fisher Scientific). Polar metabolites were extracted from the cell lysates using a modified Foch extraction as described previously [70]. 1 mL of cold 2:1 methanol:chloroform was added to 200 μL of cell lysate and vortexed to mix. The mixed solution was incubated at -80°C for 5 min, vortexed again to mix, then centrifuged for 5 min at 15,000 rpm. This yielded a biphasic solution, and 200 μL of the top polar layer was transferred in to 2 mL borosilicate glass vials and

dried in a vacuum centrifuge for gas chromatography mass spectrometry (GC-MS) analysis. To derivatize GC-MS samples prior to analysis, 20 μL of methoxyamine in pyridine (30 mg mL^{-1}) was added to each tube, sonicated in an ultrasonic bath for 1 min, and incubated for 90 min at 37°C shaking at 1000 rpm. Next, 80 μL of N-methyl-N-(trimethylsilyl)trifluoroacetamide (MSTFA) with 1% trimethylchlorosilane (TMCS) was subsequently added to each sample and the mixture was incubated at 37°C for 30 min shaking at 1000 rpm [86]. Samples and process blanks were analyzed by GC-MS (Agilent 5975C) using a HP-5MS column (30 m x 0.25 mm x 0.25 μm) with splitless injection of 1 μL of each sample and an injection port temperature of 250°C. After injection, the GC oven temperature was held at 60°C for 1 min, then ramped to 325°C at a rate of 10°C min^{-1}, followed by 5 min held at 325°C. Retention indices were calculated for detected metabolites based on analysis of a mixture of fatty acid methyl esters (FAMEs). Metabolites were identified with MetaboliteDetector [71] by matching GC-MS features to spectra and retention time indices in the Fiehn Metabolomics Retention Time Locked Library [72]. Data are reported as peak area for each feature, and features were assigned chemical names when identification was possible upon comparison to an MS library. For conversion to dry cell weight compositions, peak areas were normalized to protein concentrations measured by the BCA assay.

Additional biomass composition data were gathered from previous studies, although taxonomic coverage was less extensive for certain classes. Lipid head groups and acyl chain lengths and saturation states were only available for strain MED4 [63, 73]; amino acid profiles for strain MED4 [73], mineral content and major ions for strains NATL1A, GP2, SB, EQPAC1, SS120, and PCC 9511 [74]; iron and cobalt for strain MIT 9215 [75]. The concentrations of other vitamins, free nucleotides and nitrogenous bases, and a subset of metabolites and osmolytes not detected or not annotated in our metabolomics results have not be determined for any strain of *Prochlorococcus*; in these cases, compositions from the model cyanobacterium *Synechocystis* PCC6803 were used for all strains [45].

Pigments Initial values and boundary constraints on pigment compositions were derived from previously published datasets [4, 17, 76] for five HLII strains (MIT 9201, MIT 9202, MIT 9215, MIT 9302, MIT 9312), one HLI strain (MED4), two LLII/III strains (MIT 9211, SS120), and two LLIV strains (MIT 9303, MIT 9313). Bounds on the concentrations and

ratios of divinylchlorophyll-a, divinylchlorophyll-b, zeaxanthin, and α-carotene were collated from strains grown in batch culture or turbidostat under a range of light conditions and applied to respective ecotype strains in the acclimation pipeline (section 1.6).

Macromolecular composition Initial values and boundary constraints on macromolecular compositions were determined through nitrogen or phosphorus starvation experiments using cultures of the HLI strain MED4. Axenic cultures of MED4 (CCMP 2389) were obtained from the National Center for Marine Algae and Microbiota. All cultures were maintained at an irradiance of 60 μmol photons m^{-2} s^{-1} on a 12:12 light:dark cycle provided by cool white fluorescent bulbs and a temperature of 21°C in 4L polycarbonate bottles. Cell abundances and the purity of cultures were determined throughout all experiments using flow cytometry (BD Accuri C6) according to [77] to discriminate MED4 cells from potential contaminating heterotrophic bacteria. Nutrient-replete growth media was based on the AMP1 recipe [78] modified to have lower concentrations of ammonium (110 μM), phosphate (10 μM), and trace metals (10% of AMP1 concentrations). Nutrient-replete cultures were maintained in optically-thin semi-continuous growth and sampled for macromolecular and elemental composition when fully acclimated to these conditions (>10 generations with <15% variation in growth rate). These same parameters were assessed in separate nitrogen and phosphorus starvation experiments in which nutrient-replete cultures were diluted with N-free or P-free media (modified AMP1 media as described above without ammonium or phosphate added) and sampled four times over 7-8 days from late exponential phase to late stationary phase (4 days after cessation of growth). Samples for total particulate carbohydrate, lipid, protein, DNA, RNA, and divinyl-chlorophyll-a as well as elemental composition (particulate C, N, and P) were collected and analyzed as in [53] with the following exceptions: a) smaller pore size polycarbonate filters (0.2μm) were used to collect protein, RNA, and DNA samples; b) samples for all other macromolecules were collected on two pre-combusted glass fibre filters filters (Whatman GF/F) stacked on top of each other during filtration; and c) extracted carbohydrates were quantified using the phenol-sulfuric acid method [79]. At each sampling point, cell abundances and particulate dry weight were also determined, allowing each macromolecular pool to be expressed as a cell quota or proportion of cell mass. Cell abundances were determined as described above while samples for particulate dry weight were collected on GF/F filters as described above for macromolecular

samples and weighed after being rinsed with 0.5M ammonium formate and dried at 60°C to remove salts. The upper and lower bounds of compositions of each macromolecular pool in both nutrient starvation experiments were applied to all strains in the acclimation pipeline (described below).

1.6 Acclimation pipeline for strain GEMs

Two sequential bilevel optimizations are implemented to allow each *in silico* strain to acclimate to each environment, subject to physiological constraints (fig. S17). The first, a linear program called `OptTrans`, searches for the optimal distribution of membrane transporters and cell size, while the second, a non-linear program called PhysOpt, searches for the optimal macromolecular and pigment composition. Both optimizations repeatedly solve the FBA problem with varying compositions, using an interior point solver (Mosek 9, Mosek, API).

OptTrans It was recently shown that substrate-limited growth rates can be predicted for an arbitrary substrate using a combination of molecular modeling, quantitative proteomics, and FBA [32]. The model accounts for the dependence of both the maximum uptake rate v_{max} and the half-saturation concentration k_S on transporter abundance, cell size, and temperature. To distinguish from other reactions in the network S, we refer to a subset Q (given as subscripts q for each column) to refer to all transporter reactions, their substrates, and associated parameters. Briefly, under nutrient replete growth conditions, the substrate uptake rate ($v_q = v_{max,q}$; predicted by FBA) and transporter abundance (a_q; measured by quantitative proteomics; [80]) is used to determine the *in vivo* catalytic rate of the transporter ($k_{cat,q}$). Two additional parameters are required to compute the uptake rate dependence on transporter abundance - the effective capture area of the transporter A and the hydrated molecular diffusivity of the substrate molecule D. A_q can be quantified by measuring the dimensions (PyMol; The PyMOL Molecular Graphics System, Version 2.3 Schrodinger, LLC) of the transmembrane domain of each transporter using 3-dimensional renderings of the predicted protein structure (RaptorX, [81]). D_q is quantified as an empirical function of hydrated molecular volumes ([82]; using the LeBas incremental method; [83]) and the dynamic viscosity, determined as a function of *in situ* temperature, pressure, and salinity. Given a known temperature and bulk concentration of substrate S_q^{∞}, these three parameters are sufficient to calculate the effective half saturation concentrations at two limits - the diffusive limit $k_{s,q}^{D}$ as $S_q^{\infty} \to 0$, which is independent of the number of transporters, and the so-called porter limit $k_{s,q}^{P}$ as $S_q^{\infty} \to \infty$), which is dependent on the number of transporters. The uptake rate v_q is then

$$v_q = \frac{v_{max,q} S_q^\infty}{k_{s,q}^P + k_{s,q}^D + S_q^\infty}. \tag{13}$$

This transport model was applied in an optimization framework, termed `OptTrans`, to account for multiple substrates and to allow for flexibility in cell size. Cell size and transporter density (transporters m^{-2}) for each substrate (e.g., nitrate, nitrite, ammonium, phosphate) are optimized, subject to membrane surface area constraints, transporter catchment areas, and *in vivo* catalytic rates. Cells are assumed to be spherical and we do not currently optimize the shape coefficient ϕ. The Sherwood number is also not explicitly considered, and we assume transport due to advective shear to be equal to that of molecular diffusion within the boundary layer of the cell. With these two simplifications, the bi-level optimization for `OptTrans` includes an outer program which varies the number of transporters a and the cell size r and assigns flux bounds to corresponding transport reactions in the inner program. Let $a \in \mathbb{R}^Q$ be a vector with elements a_q that comprise the number of transporters for substrate q, and let r be the radius of the cell. Then `OptTrans` maximizes the objective $\mu(a, r)$ as follows:

$$\begin{aligned}
\underset{a \in \mathbb{R}^Q, r \in \mathbb{R}}{\text{maximize}} \quad & \mu(a, r) \\
\text{subject to} \quad & \arg\max_{v \in \mathbb{R}^n} \mu = c^T v, \text{ s.t. } Sv = 0,\ v_j^{lb} \leq v_j \leq v_j^{ub}, \\
& v_q^{lb} = v_q^{ub} = \frac{a_q k_{cat,q} S_q^\infty}{k_{s,q}^P + k_{s,q}^{D_q} + S_q^\infty}, \\
& k_{s,q}^D = \frac{a_q k_{cat,q}}{\phi Sh} D_q r, \\
& k_{s,q}^P = \sqrt{\frac{A_q}{\pi}} \frac{\pi k_{cat,q}}{4 \alpha_q A_q D_q \sqrt{A_q \pi}}, \\
& \alpha_q = \frac{\nu_q \sqrt{A_q \pi}}{4 D_q}, \\
& \nu_q = \frac{D_q}{r} + \frac{u}{2}, \\
& \sum_{q=1}^{n} A_q a_q \leq f 4 \pi r^2, \\
& \forall \{q \in Q, Q \subseteq n\}
\end{aligned} \tag{14}$$

For the AMT simulations, different strains are capable of transporting different nutrients (ammonia, nitrite, nitrate, and phosphate). For strains lacking genomic or experimental evidence of a particular transporter, the upper bound on its abundance is set to zero.

The optimal transporter abundance a^* and a critical substrate concentration S^* were calculated [32] as a reference for comparison against simulated optima. S^* represents the delineation between two rate limiting regimes - diffusion limitation ($S \leq S^*$), wherein a^* is the minimal number of transporters required to maintain uptake rates at the diffusive flux, and growth limitation ($S > S^*$), wherein a^* is the minimal number of transporters required to maintain maximal growth, set by some other limiting factor (e.g., growth limitation by another resource, ribosome limitation of translation rates, RuBP carboxylase capacity, molecular crowding, etc.). When used in conjunction with other metrics of nutrient stress (e.g., metabolite sensitivity analysis), comparisons of ambient nutrient concentrations with S^* and comparisons of optimal a with a^* can parse otherwise indistinguishable nutrient limitation states.

PhysOpt Uptake rates from `OptTrans` are set as upper bounds on transporter fluxes for the second bilevel optimization step, `PhysOpt`, which searches for the optimal macromolecular composition and pigment composition for each strain in each grid cell, wherein the spectral light field and the nutrient supply are known. Additionally, the optimal cell radius r from `OptTrans` is implemented in `PhysOpt` as cell ash-free dry weights (M; g DW cell^{-1}), converted from the radius as described previously.

Similar to `OptTrans`, the outer program of `PhysOpt` solves for elements of the constraints on the vector of fluxes v corresponding to the absorption reactions in the inner program. However, in contrast to `OptTrans`, `PhysOpt` also simultaneously adjusts coefficients of the S matrix corresponding to the biomass reaction (denoted by the column superscript B). These compositions are subject to experimentally determined upper and lower bounds on each macromolecular pool i and subject to the simplex constraint ($\sum_{i=1}^{m} S_i^B = 1$). Since each pigment and macromolecular pool incurs a different elemental and energetic cost, tradeoffs in resource allocation are implicitly considered as part of the holistic fitness maximization. For example, *de novo* protein synthesis is energetically costly relative to total biomass, so it might be expected that compositions would be depleted at low light levels, however since more

than 90% of total cellular nitrogen is bound up in protein, the fitness cost of high protein content in the nitrogen-deplete surface layer is considerably higher. Note that, in principle, the `PhysOpt` formulation need not be restricted to such a coarse resolution (e.g., protein) and could accommodate metabolomic datasets (i.e., individual amino acids).

Several reactions corresponding to the absorption of light by each pigment, as well as reactions representing the transitions through excitation and relaxation of electronic states of each pigment were added. To distinguish between these and other reactions, and for clarity, we refer to this subset using the subscript p. Thus, in `PhysOpt`, the rate of each pigment absorption reaction v_p corresponds to the wavelength-integrated product of the pigment's specific absorption spectrum a_p^* [84], the downwelling irradiance spectrum E_d, and the amount of pigment g_p. Irradiance and absorption data typically spanned the range $(\lambda_{min} : \lambda_{max})$ from 350 nm to 800 nm. In addition to the experimentally determined upper and lower bounds on g_p (g_p^{ub} and g_p^{lb}, respectively), we also include the upper and lower bounds on the divinylchlorophyll b/a ratio $\frac{g_{dvChlb}}{g_{dvChla}}$ ($g_{b/a}^{ub}$ and $g_{b/a}^{lb}$, respectively) as a further stoichiometric constraint on pigment compositions. The packaging effect is not currently included, as it should not appreciably influence absorption for cells as small as *Prochlorococcus*. The full `PhysOpt` formulation is:

$$
\begin{aligned}
\underset{S_i^B \in \mathbb{R}^m}{\text{maximize}} \quad & \mu(S_i^B) \\
\text{subject to} \quad & \arg\max\, Z = c^T v, \text{ s.t. } Sv = 0,\ v^{lb} \le v \le v^{ub}, \\
& a_p = \int_{\lambda_{min}}^{\lambda_{max}} g_p E_d a_p^* d\lambda, \\
& g_p^{lb} \le g_p \le g_p^{ub}, \\
& g_{b/a}^{lb} \le \frac{g_{dvChlb}}{g_{dvChla}} \le g_{b/a}^{ub}, \\
& S_i^B = \frac{g_p}{\frac{4}{3}\pi r^3 M}, \\
& S_i^{B,lb} \le S_i^B \le S_i^{B,ub}, \\
& \sum_{i=1}^{m} S_i^B = 1, \\
& \forall \{i \subseteq m, j \subseteq n, p \in i\}
\end{aligned}
\tag{15}
$$

1.7 Temperature dependence of metabolic rates

Growth rates and metabolic fluxes were adjusted for temperature effects by comparing *in situ* growth temperatures to a model of temperature dependent growth, parameterized for each strain. Our approach provides a strain-specific parameterization while avoiding the effects of adaptive laboratory evolution, which can be substantial [55]. First, the Arrhenius activation energy E_a was calculated based on growth rates determined in batch cultures maintained over a range of temperatures T (°K; fig S15) [9, 56],

$$ln\,\mu = ln\,A - \frac{E_a}{RT} \tag{16}$$

where A is a dimensionless constant and R is the ideal gas constant (8.314 J mol^{-1} K^{-1}). Calculated activation energies were statistically identical for the 12 strains (p=0.415) with a mean value of 52.7±5.1 KJ mol^{-1} (R^2 = 0.89; p = 5.1 × 10^{-20}).

The Arrhenius function captures the temperature dependence of growth rate within a range where enzyme stability is preserved. At temperatures exceeding that range, growth rates of the 12 analyzed strains rapidly approached zero, requiring two additional parameters (optimal growth temperature T^* and maximum growth temperature T^+) to capture these dynamics. We could detect no consistent pattern in the width of the interval between T^* and T^+, so a single parameterization (2±0.8°C above T^*) was assumed for all strains. A modification of a previously proposed model [57], but which preserves the Arrhenius parameterization, was used to capture the dynamics of a dimensionless growth rate constant k in both regimes (fig. S14c),

$$k = e^{\frac{-E_a}{RT}}(1 - e^{T-T^+}). \tag{17}$$

Optimal growth temperatures (OGT; T^*) were predicted for each strain from proteome sequences using the machine learning algorithm TOME-cool. The OGT dataset from TOME 1.0 [26] was expanded to include additional psychrophilic and psychrotolerant taxa [58, 59], resulting in a training dataset of 6020 microorganisms (`https://github.com/EngqvistLab/tome_cool`). The distribution of OGT values is shown in fig. S18. The same machine learning approach as described in [26] which uses 5-fold cross-validation with 5-fold inner cross-validation for hyperparameter optimization was used to select the best regression model. Among linear, elastic net, Bayesian Ridge, decision tree, and random forest regression models, the support vector machine regression achieves the best performance (coefficient of determination

score, $R^2 = 0.88$ obtained from the cross-validation, fig. S19). The residual plots show that the model tends to overestimate the OGT values for psychrophiles (fig. S19b). As the OGT values shows a skewed distribution with most samples with an OGT between 20°C and 40°C, we next binned OGT values with an interval size of 5°C. Equal weights were assigned to each bin. Organism-specific weights were calculated by normalizing each bin weight by the number of organism in each bin. The weighted regression was used, and different regression models were tested with the same cross-validation approach. Among those, the Bayesian Ridge regression showed the best performance ($R^2 = 0.65$, fig. S19c). The residual plot shows that the model improved performance for psychrophiles, however at the cost of precision for other organisms (fig. S19d). The fitting results with the best models (SVR and Bayesian Ridge) from those two different settings was compared (fig. S19e-g).

1.8 Weighted population rates and stocks

Acclimated single cell biomass-specific fluxes and compositions were scaled up to whole population, volumetric rates and concentrations (stocks) using qPCR based ecotype abundance data [9]. Since the primers captured a variable proportion (75 ± 21%) of the total *Prochlorococcus* FCM counts, ecotype relative abundances were scaled to flow cytometric cell counts, thereby treating the unknown remaining population as an average of those quantified. Rather than assuming an even composition of strains within each ecotype, the most fit strain in each grid cell was chosen as a representative. Accordingly, population variables X_{pop} (e.g., metabolic fluxes, macromolecular compositions, elemental stoichiometries) were calculated as an abundance weighted sum of a variable associated with the fittest strains from each ecotype x_e,

$$X_{pop} = N_{FCM} \sum_{e=1}^{E} b_e x_e M_e \tag{18}$$

where B_{FCM} is the flow cytometry derived total *Prochlorococcus* cell count, b_e is the relative abundance of the eth ecotype in the set of all ecotypes E, and M_e is the ash-free dry weight of cellular biomass for the most fit strain within each ecotype, approximated using its calculated optimal cell size and a fixed dry weight content of 21% and ash content of the dry weight of 16% [73].

2 Datasets

Data S1 - Strains (data_S1.csv) Table of *Prochlorococcus* strains included in this manuscript and a variety of metadata associated with each strain. Columns include link identifiers to NCBI and IMG databases, genome metrics and statistics, sequencing information, taxonomic classifications, cell size and maximum photosynthetic rates.

Data S2 - AMT-13 cruise data (data_S2.csv) Table of 66 variables gridded at 1m depth resolution with associated coordinates, timestamps, and transect station names. Data provided with permission from the BODC.

Data S3 - Optimal growth temperatures (data_S4.csv) Table of optimal growth temperatures predicted by TOME for each of the 69 isolate genomes included in simulations.

Data S4 - Macromolecular and elemental compositions (data_S5.xlsx) Tables of results from experiments with strain MED4 for the determination of macromolecular compositions across a range of nitrogen (ammonia) and phosphate stress growth phases. Data include the macromolecular compositions of biomass (DNA, RNA, protein, lipid, pigments, divinylchlorophyll a, phospholipid, carbohydrate, and polyphosphate), the dry weight of cells, nutrient concentrations of the media at the time of harvest, cell counts, and elemental compositions (C, N, and P). Three replicate samples are reported for each sampling time point. Details of the experimental design and methods are also included.

Data S5 - Metabolomics (data_S6.csv) Metabolomics data for strains from each of the five ecotypes (MED4, MIT 9312, NATL2A, MIT 1304, and MIT 9313). Data and summary statistics for 5 replicate samples of each strain are included. Data are reported in m/z peak area for each feature, and features were assigned chemical names when possible.

Data S6 - Literature sources (data_s7.xlsx) Table of literature sources for parameters used in simulations, and for which strains data were available.

3 Tables

Strain	Ecotype	# rxns	# mets	# genes
PanGEM		1484	1282	1117
EQPAC1	HLI	1112	1003	842
MED4	HLI	1111	1001	842
MIT9515	HLI	1105	982	828
AS9601	HLII	1100	995	824
GP2	HLII	1113	979	838
MIT0604	HLII	1107	993	838
MIT1314	HLII	1102	990	832
MIT9107	HLII	1106	993	831
MIT9116	HLII	1105	991	831
MIT9123	HLII	1106	993	831
MIT9201	HLII	1089	980	816
MIT9202	HLII	1082	982	827
MIT9215	HLII	1105	995	838
MIT9301	HLII	1106	997	826
MIT9302	HLII	1111	1001	838
MIT9311	HLII	1122	993	844
MIT9312	HLII	1122	993	844
MIT9314	HLII	1113	1000	844
MIT9321	HLII	1100	989	826
MIT9322	HLII	1100	989	826
MIT9401	HLII	1100	989	826
RS01	HLII	1106	992	833
RS04	HLII	1106	992	833
RS50	HLII	1106	992	833
SB	HLII	1101	986	832
XMU1401	HLII	1096	985	833
MIT0801	LLI	1123	992	850
MIT0912	LLI	1124	993	845
MIT0913	LLI	1125	994	845
MIT0915	LLI	1129	1000	851
MIT0917	LLI	1127	1003	850
MIT1013	LLI	1138	1004	850
MIT1214	LLI	1133	1011	856
NATL1A	LLI	1131	999	856
NATL2A	LLI	1128	998	851
PAC1	LLI	1123	992	850
LG	LLII/LLIII	1114	997	834
MIT0601	LLII/LLIII	1085	994	815
MIT0602	LLII/LLIII	1119	1003	840
MIT0603	LLII/LLIII	1120	1005	840
MIT0918	LLII/LLIII	1125	1005	852
MIT0919	LLII/LLIII	1085	994	815
MIT1304	LLII/LLIII	1132	1018	855
MIT9211	LLII/LLIII	1104	991	829
SS120	LLII/LLIII	1117	999	835
SS2	LLII/LLIII	1115	999	834
SS35	LLII/LLIII	1114	997	834
SS51	LLII/LLIII	1114	997	834
SS52	LLII/LLIII	1114	997	834
MIT0701	LLIV	1151	1020	875
MIT0702	LLIV	1152	1022	875
MIT0703	LLIV	1151	1020	875
MIT1205	LLIV	1149	1020	877
MIT1227	LLIV	1151	1024	876
MIT1303	LLIV	1156	1020	879
MIT1306	LLIV	1158	1024	880
MIT1312	LLIV	1153	1027	878
MIT1313	LLIV	1159	1026	882
MIT1318	LLIV	1159	1026	882
MIT1320	LLIV	1156	1025	880
MIT1323	LLIV	1155	1026	878
MIT1327	LLIV	1153	1027	878
MIT1342	LLIV	1165	1035	888
MIT1418	LLIV	1157	1026	880
MIT9303	LLIV	1170	1041	886
MIT9313	LLIV	1157	1024	881
P1344	LLIV	1151	1024	876
P1361	LLIV	1153	1027	878
P1363	LLIV	1153	1027	878

Parameter	Description	Value	Units
α	Substrate molecule capture probability	Mod.	dimensionless
δ	FVA tolerance	Mod.	dimensionless
$\Delta_f G'^o$	Gibbs energy of formation	Mod.	J mol^{-1}
ΔH_C	Enthalpy of combustion	Mod.	J mol^{-1}
γ	Shadow price	Mod.	h^{-1}
λ	Wavelength	Obs.	nm
$\lambda_{1,2}$	Dual variables	Mod.	dimensionless
μ	Growth rate	Mod.	s^{-1}
ν^D	Diffusive molecular encounter velocity	Mod.	m s^{-1}
ν^P	Membrane transport velocity at the porter limit	Mod.	m s^{-1}
ϕ	Shape coefficient	1	dimensionless
a_p	Pigment specific absorption	Mod.	mmol gDW^{-1} s^{-1}
a_{ij}	Stoichiometric coefficient	Mod.	dimensionless
A	Transporter capture area	Molec.	m^2
b_e	Ecotype relative abundance	Obs.	cells ml^{-1}
c	Objective weighting vector	Mod.	dimensionless
D	Hydrated molecular diffusivity	Molec.	m^2 s^{-1}
E_a	Arrhenius activation energy	Obs.	J mol^{-1}
E_d	Downwelling irradiance	Obs.	W m^{-2} nm^{-1}
f	Objective function value	Mod.	h^{-1}
f_{max}	Maximum fraction of cell surface area for transporters	0.085	dimensionless
g_p	Pigment coefficient	Obs.	g gDW^{-1}
k_{cat}	Catalytic rate	Mod.	s^{-1}
k_d	Spectral attenuation	Obs.	m^{-1}
$k_j^{\pm}$	Reaction rate constant	Mod.	s^{-1}
k_s	Half-saturation concentration	Mod.	mol m^{-3}
k_s^P	Half-saturation concentration at the porter limit	Mod.	mol m^{-3}
k_s^D	Half-saturation concentration at the diffusive limit	Mod.	mol m^{-3}
k_{cat}	Catalytic rate of an individual transporter	Mod.	mol s^{-1}
l^R	Reaction-diffusion characteristic length scale	Mod.	m
M	Cell ash-free dry weight	Obs.	gDW cell^{-1}
n	Number of transporters	Mod.	cell^{-1}
n_{max}	Maximum number of transporters	Mod.	cell^{-1}
n_G	Number of transporters of replete batch-acclimated cells	Exp.	cell^{-1}
n^*	Optimal number of transporters for any phenotype	Mod.	cell^{-1}
n_G^*	Optimal number of transporters at the growth limit	Mod.	cell^{-1}
n_D^*	Optimal number of transporters at the diffusion limit	Mod.	cell^{-1}
Q	Cellular biomass quota	Mod.	mol X cell^{-1}
r	Cell radius	Mod.	m
R	Ideal gas constant	Mod.	J mol^{-1} K^{-1}
S	Stoichiometric matrix	Mod.	dimensionless
S_∞	Ambient substrate concentration	Obs.	mol m^{-3}
S^*	Nutrient limitation concentration	Mod.	mol m^{-3}
S_G^{lb}	Lower bound of the n_G^* feasible domain	Mod.	mol m^{-3}
S_{SA}^{lb}	Lower bound of surface area limitation	Mod.	mol m^{-3}
S_{SA}^{ub}	Upper bound of surface area limitation	Mod.	mol m^{-3}
Sh	Sherwood number	1	dimensionless
T	Temperature	Obs.	K
T^*	Optimal growth temperature	Mod.	K
T^+	Maximum growth temperature	Mod.	K
u	Advective velocity	Obs.	m s^{-1}
v	Flux	Mod.	mol s^{-1}
v_{lb}	Lower flux bound	Mod.	mmol gDW^{-1} h^{-1}
v_{ub}	Upper flux bound	Mod.	mmol gDW^{-1} h^{-1}
v_{max}	Maximum flux	Mod.	mol s^{-1}
v_{max}^G	Maximum flux of replete batch-acclimated cells	Mod.	mol s^{-1}
V	Cell volume	Obs.	m^3
x_i	Concentration	Mod.	M
X_{pop}	Population weighted average value	Mod.	various
$Y_{X/S}$	Biomass yield	Mod.	mol X [mol S]$^{-1}$
z	Depth	Obs.	m

4 Supplemental figure captions

Fig. S1 *Pangenome orthologs.* Left - Matrix of KEGG ortholog (KO) presence for each strain. KO's associated with strains included in the AMT-13 simulation are indicated in red, those not included are indicated in green. Columns are ordered by the total number of KO's associated with each strain, rows are ordered by the total number of strains associated with each KO. Corresponding genome sizes are shown above. Right - Rarefaction curves for the pangenome (top) and the core subset (bottom). Mean and standard deviations were computed from 100 iterations.

Fig. S2 *Network similarity.* Matrix of Jaccard similarity for all strain included in the AMT-13 simulation. Strains within each ecotype are ordered by their within-ecotype aggregate rank similarity.

Fig. S3 *Cruise Variables I: Physics.* Meridional sections of potential temperature (top left), salinity (top right), potential density (bottom left), and photosynthetically active radiation (PAR; log scale; bottom right).

Fig. S4 *Cruise Variables II: Nutrients.* Meridional sections of ammonium (top left), nitrite (top right), nitrate (bottom left), and phosphate (bottom right). All data plotted on a log scale.

Fig. S5 *Cruise Variables III: Biology.* Meridional sections of fluorometric chlorophyll a, *Prochlorococcus*, *Synechococcus*, picoeukaryotic phytoplankton, nanoeukaryotic phytoplankton, Coccolithophores, Cryptophytes, and heterotrophic bacteria.

Fig. S6 *Ecotype growth rate depth profiles.* Selected depth profiles of predicted ecotype growth rates along the AMT-13 cruise track. Profiles are shown for 28°S, 1°N, and 38°N. Panels in this figure are replotted from fig. 2e-i in the main text to highlight differences in the depth structure which are difficult to discern in contour plots.

Fig. S7 *Primary production.* Top panel - Comparison of predicted and observed depth-integrated (5-200 m) rates of primary production. ^{14}C-PP data were acquired during roughly 12 hour incubations and most closely approximate net primary production (NPP). By accounting for respiratory oxygen consumption during 24 hour incubations in paired dark bottles, Winkler-GPP most closely approximates gross primary production. Both incubations integrate the entire microbial community. Pro-NPP is defined by the rate of inorganic carbon exchange (uptake minus loss) and is comparable with

^{14}C-PP data; Pro-GPP is the rate of oxygen produced by photosystem II and is comparable with Winkler-GPP. Bottom panel - Meridional section of *Prochlorococcus* GPP.

Fig. S8 *f-ratio.* Meridional section of predicted f-ratio (the fraction of total nitrogen uptake supplied by nitrate) for the total *Prochlorococcus* population.

Fig. S9 *Elemental stoichiometry.* Meridional sections of population average carbon : nitrogen (top panel), carbon : phosphorus (middle panel), and nitrogen : phosphorus (bottom panel) elemental ratios.

Fig. S10 *Entalpy of combustion of biomass.* Meridional sections of computed optimal enthalpy of combustion (KJ gDW^{-1}) of biomass for each of the five ecotypes.

Fig. S11 *Fitness gains due to acclimation.* The effect of allowing for acclimation is shown in terms of absolute growth rate (top panel) and the relative increase in growth rate (bottom panel). Fitness gains are shown for each of the bilevel optimization steps (`OptTrans` and `PhysOpt`). Simulations without acclimation were initialized with the optimal laboratory growth physiological parameters and not subjected to optimization. The number of observations from each subset are not equal due to an unequal number of infeasible growth realizations.

Fig. S12 *Cell size.* Meridional section of population average optimal carbon quotas.

Fig. S13 *Steady-state critical ammonia concentrations.* Top panel - meridional section of the critical concentration S^* of ammonia for the *Prochlorococcus* population. Bottom panel - histograms of ambient ammonia concentrations (blue bars) and predicted S^* (red bars) across the transect.

Fig. S14 *Divinylchlorophyll a/b ratios.* Top panel - Correspondence of divinylchlorophyll a/b ratios (moles divinylchlorophyll b per mole divinylchlorphyll a) to the downwelling irradiance spectral band ratio of 474 nm to 450 nm (moles 474 nm photons m^{-2} s^{-1} per mole 450 nm photons m^{-2} s^{-1}). These bands correspond to the spectral absorption peaks of divinylchlorophylls b and a, respectively. The $\frac{E_d(474)}{E_d(450)}$ ratio typically increases with depth in the water column. Bottom panel - meridional section of average divinylchlorophyll b/a ratios for the *Prochlorococcus* population.

Fig. S15 *Temperature dependence.* Left panel - growth rates of 12 strains of *Prochlorococcus*, each grown over a range of cultivation temperatures. Supra-optimal growth temperatures are excluded from this plot. Least-squares regression line is shown, corresponding to an activation energy of 52.7±5.1 KJ mol^{-1}. Middle panel - violin plots of TOME-cool predicted optimal growth temperatures for each of the five ecotypes analyzed. Right panel - comparison of the observed and predicted temperature dependence of growth rates for two strains (MED4 and MIT 9312). Predictions are based on TOME-cool OGT parameterizations of our modified Arrhenius function for each strain.

Fig. S16 *Growth capabilities.* Matrix of growth/no growth capabilities for the PanGEM (leftmost column) and each of the 69 strains simulated. Strains are grouped within their respective ecotypes (colors). Dark shading indicates that the corresponding strain is capable of growth on the corresponding substrate as a sole nitrogen or phosphorus source.

Fig. S17 *OptTrans and PhysOpt schema.* A - The OptTrans algorithm optimizes each of the q transport reactions in an outer program, assigning corresponding elements of v (blue font). At each iteration of the outer program, the inner program optimizes the remaining elements of v (red font). All elements of the stoichiometric matrix S are fixed (purple font). B - In PhysOpt, both the column of S corresponding to the biomass formation reaction and the elements of v corresponding to the p pigment absorption reactions are optimized in the outer program. Similarly to OptTrans, at each iteration of the outer program, the inner program optimizes the remaining elements of v.

Fig. S18 *TOME training set distribution.* Distribution of optimal growth temperatures (OGT) of the training set (6020 organisms). Data were binned in 2°C intervals.

Fig. S19 *Summary of OGT prediction with weighted regression.* (a) Comparison between predicted and experimental OGT values from SVR model in the cross-validation. (b) Residual plot comparing the differences between predicted and experimental values in the cross-validation. (c-d) Same as in (a-b), but with weighted regression using the Bayesian Ridge linear model. (e) Fitting results for the final SVR model, without sample weights, corresponding to the cross-validation results in (a). (f) Fitting results for the final Bayesian Ridge model, with sample weights, corresponding to the cross-validation results in (c). (g) Residual plot comparing SVR and

Bayesian Ridge strategies. Lines correspond to residual values in an interval of 5°C; shaded regions indicate the 95% confidence intervals.

5 Supplemental figures

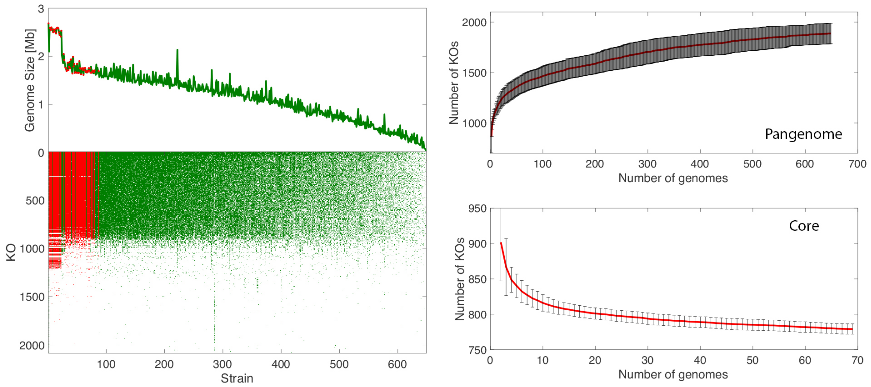

Fig. S1

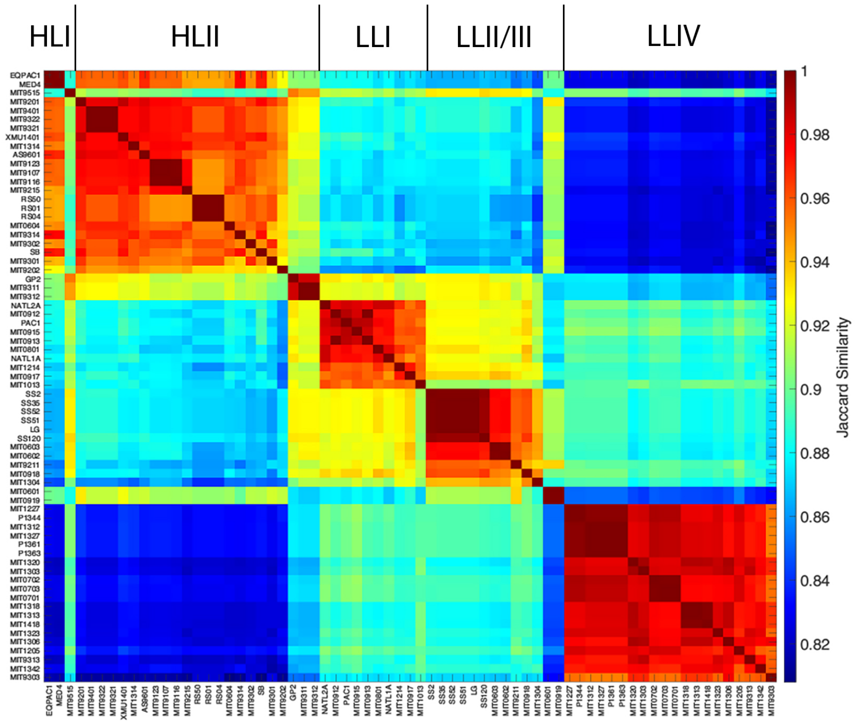
HLI
HLII
LLI
LLII/III
LLIV
Jaccard Similarity
1
0.98
0.96
0.94
0.92
0.9
0.88
0.86
0.84
0.82

Fig. S2

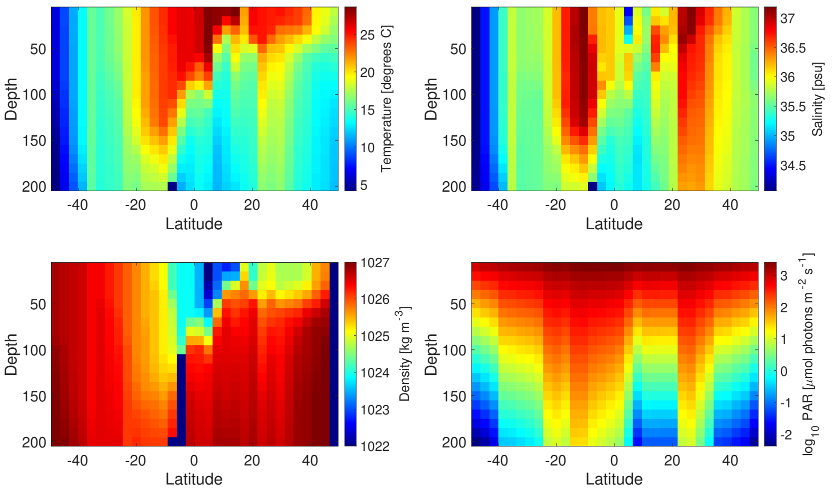
Depth
Latitude
Temperature [degrees C]
Salinity [psu]
Density [kg m^-3]
log10 PAR [μmol photons m^-2 s^-1]

Fig. S3

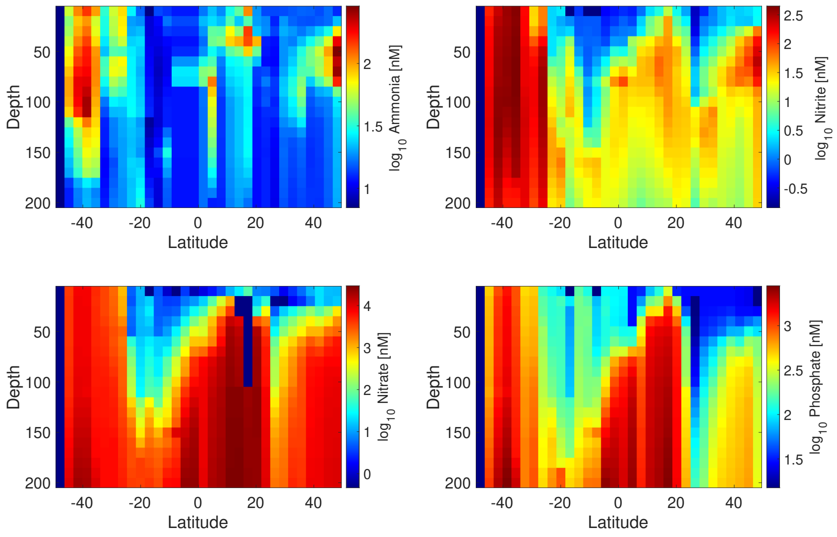

50
100
150
200
Depth
-40
-20
0
20
40
Latitude
1
1.5
2
log10 Ammonia [nM]
-0.5
0
0.5
1
1.5
2
2.5
log10 Nitrite [nM]
0
1
2
3
4
log10 Nitrate [nM]
1.5
2
2.5
3
log10 Phosphate [nM]

Fig. S4

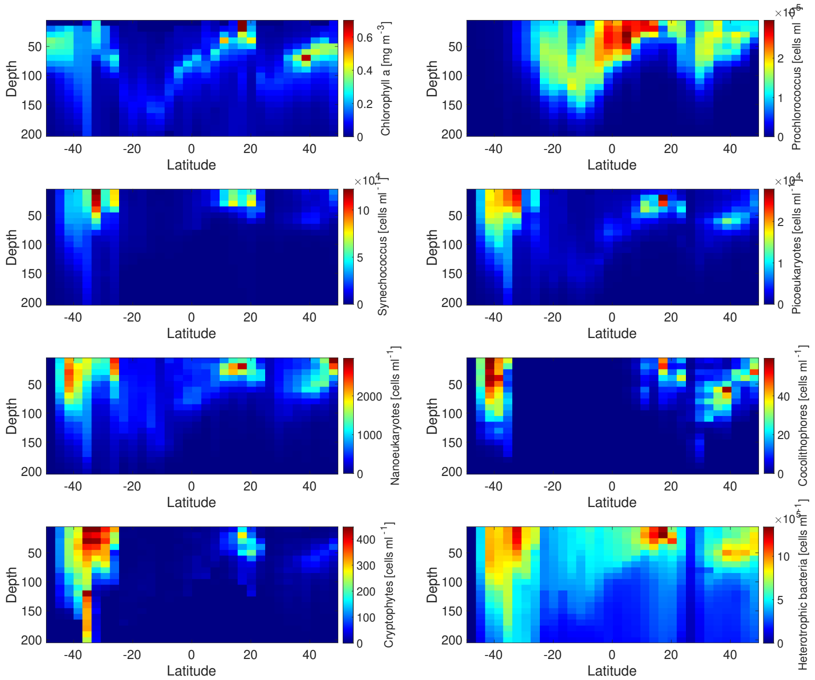

Depth
Latitude
Chlorophyll a [mg m⁻³]
Prochlorococcus [cells ml⁻¹]
Synechococcus [cells ml⁻¹]
Picoeukaryotes [cells ml⁻¹]
Nanoeukaryotes [cells ml⁻¹]
Cocolithophores [cells ml⁻¹]
Cryptophytes [cells ml⁻¹]
Heterotrophic bacteria [cells ml⁻¹]

Fig. S5

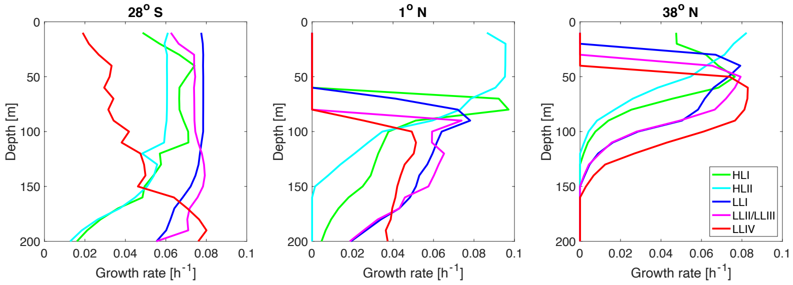

Fig. S6

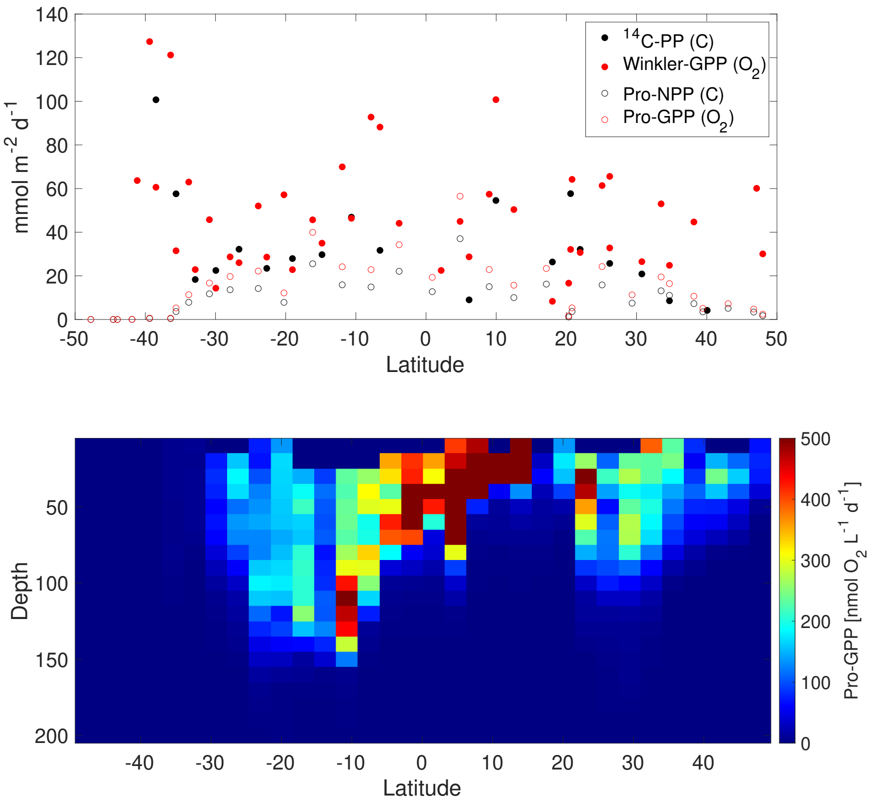

140
120
100
80
60
40
20
0
mmol m-2 d-1
-50
-40
-30
-20
-10
0
10
20
30
40
50
Latitude
14C-PP (C)
Winkler-GPP (O2)
Pro-NPP (C)
Pro-GPP (O2)
50
100
150
200
Depth
Latitude
Pro-GPP [nmol O2 L-1 d-1]
500
400
300
200
100
0

Fig. S7

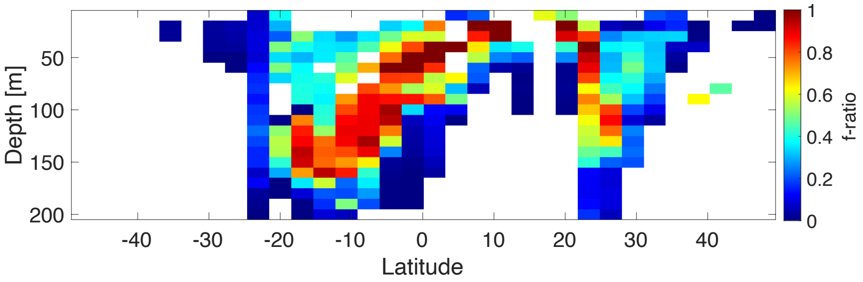

Depth [m]
50
100
150
200
-40
-30
-20
-10
0
10
20
30
40
Latitude
f-ratio
1
0.8
0.6
0.4
0.2
0

Fig. S8

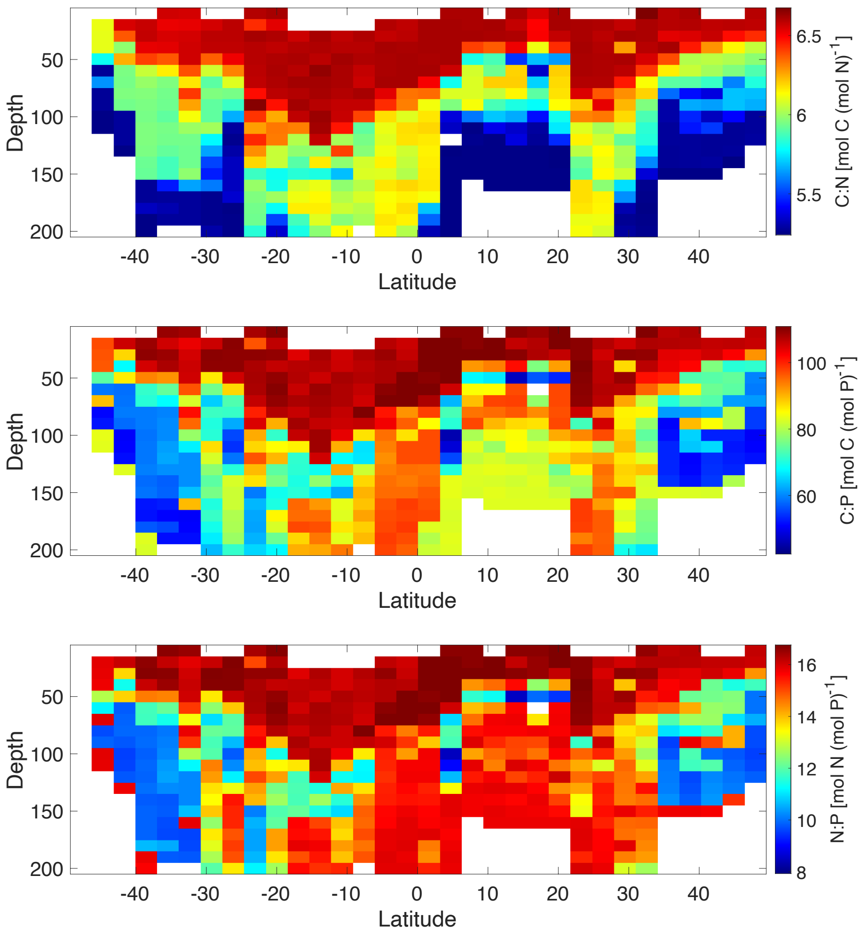

Depth
50
100
150
200
-40
-30
-20
-10
0
10
20
30
40
Latitude
C:N [mol C (mol N)$^{-1}$]
6.5
6
5.5
Depth
50
100
150
200
-40
-30
-20
-10
0
10
20
30
40
Latitude
C:P [mol C (mol P)$^{-1}$]
100
80
60
Depth
50
100
150
200
-40
-30
-20
-10
0
10
20
30
40
Latitude
N:P [mol N (mol P)$^{-1}$]
16
14
12
10
8

Fig. S9

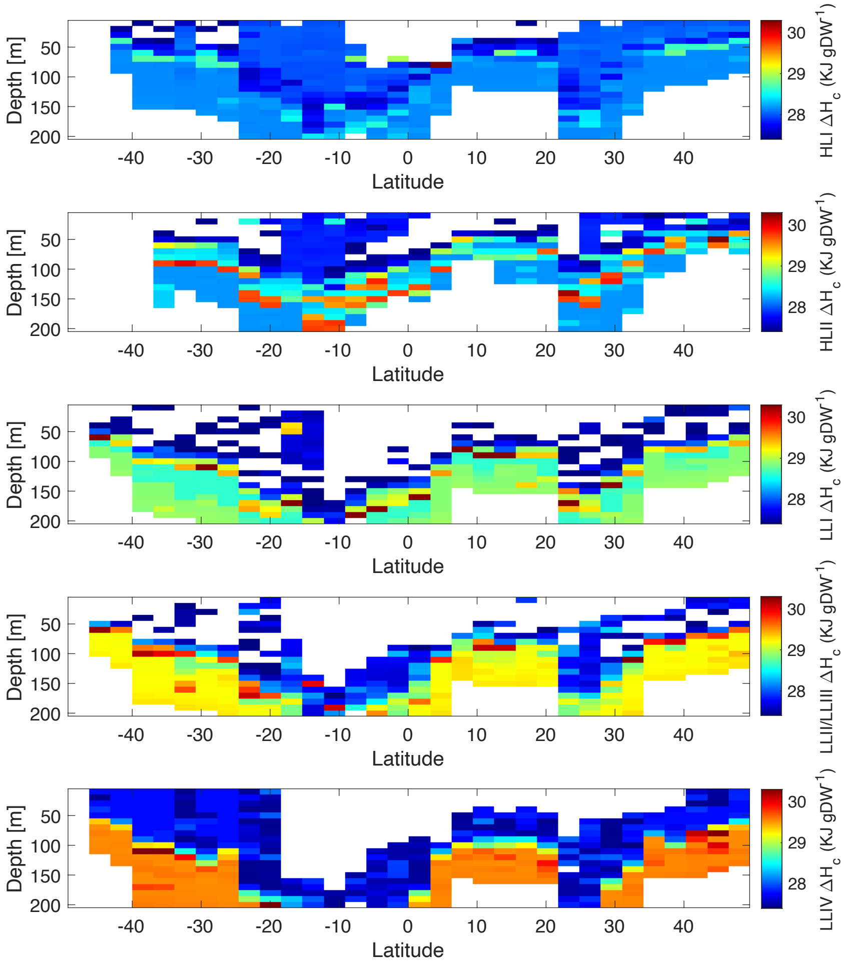

Depth [m]
Latitude
HLI ΔH$_c$ (KJ gDW^{-1})
HLII ΔH$_c$ (KJ gDW^{-1})
LLI ΔH$_c$ (KJ gDW^{-1})
LLII/LLIII ΔH$_c$ (KJ gDW^{-1})
LLIV ΔH$_c$ (KJ gDW^{-1})

Fig. S10

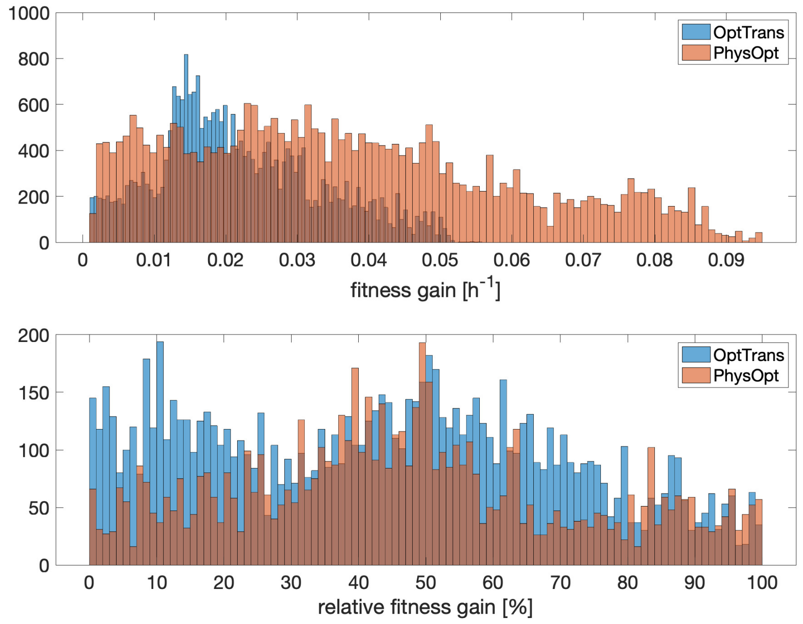

OptTrans
PhysOpt
fitness gain [h^{-1}]
OptTrans
PhysOpt
relative fitness gain [%]

Fig. S11

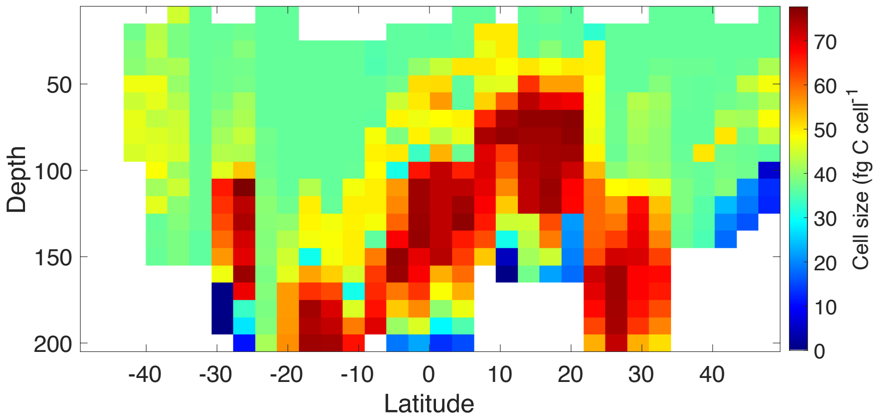
Depth
50
100
150
200
-40
-30
-20
-10
0
10
20
30
40
Latitude
Cell size (fg C cell^{-1})
70
60
50
40
30
20
10
0

Fig. S12

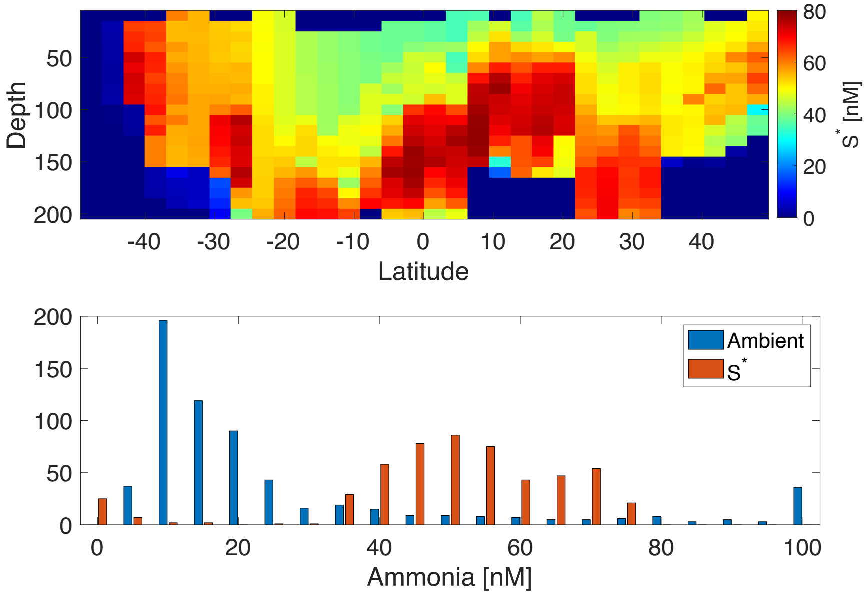

Depth
50
100
150
200
-40
-30
-20
-10
0
10
20
30
40
Latitude
80
60
40
20
0
S* [nM]
200
150
100
50
0
Ambient
S*
0
20
40
60
80
100
Ammonia [nM]

Fig. S13

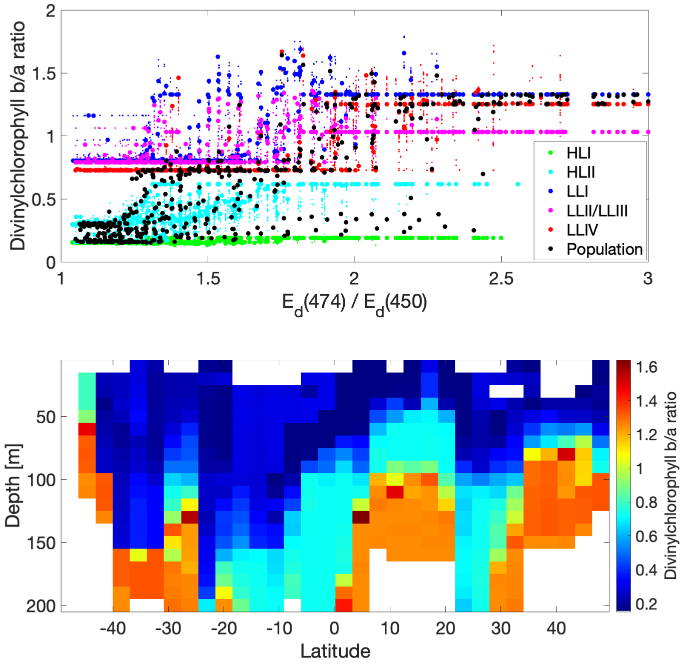

Divinylchlorophyll b/a ratio
$E_d(474) / E_d(450)$
HLI
HLII
LLI
LLII/LLIII
LLIV
Population
Depth [m]
Latitude
Divinylchlorophyll b/a ratio

Fig. S14

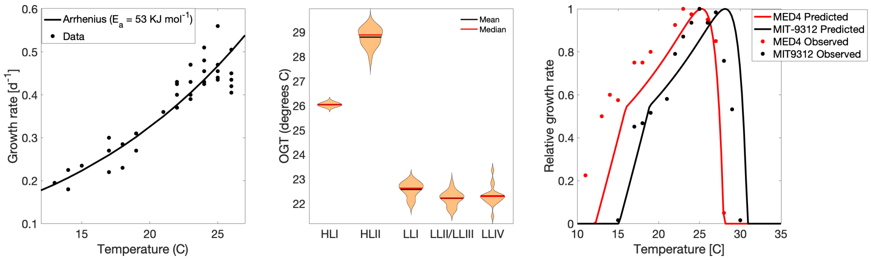
Arrhenius (E_a = 53 KJ mol^{-1})
Data
Growth rate [d^{-1}]
Temperature (C)
Mean
Median
OGT (degrees C)
HLI
HLII
LLI
LLII/LLIII
LLIV
MED4 Predicted
MIT-9312 Predicted
MED4 Observed
MIT9312 Observed
Relative growth rate
Temperature [C]

Fig. S15

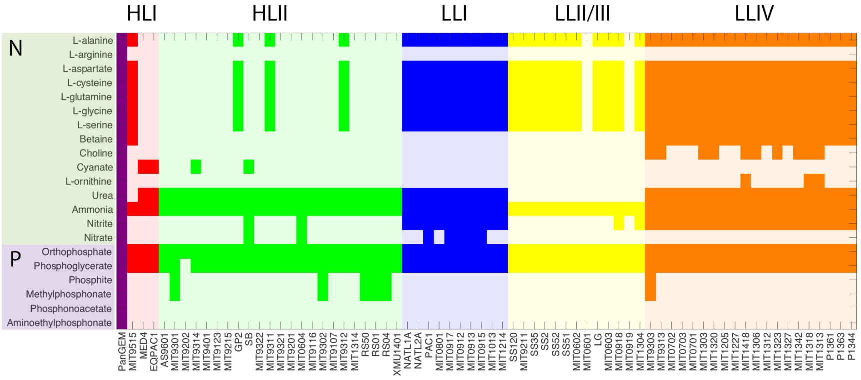

HLI
HLII
LLI
LLII/III
LLIV
N
P
L-alanine
L-arginine
L-aspartate
L-cysteine
L-glutamine
L-glycine
L-serine
Betaine
Choline
Cyanate
L-ornithine
Urea
Ammonia
Nitrite
Nitrate
Orthophosphate
Phosphoglycerate
Phosphite
Methylphosphonate
Phosphonoacetate
Aminoethylphosphonate
PanGEM
MIT9515
MED4
EQPAC1
AS9601
MIT9301
MIT9202
MIT9314
MIT9401
MIT9123
MIT9215
GP2
SB
MIT9322
MIT9311
MIT9321
MIT9201
MIT0604
MIT9116
MIT9302
MIT9107
MIT9312
MIT1314
RS50
RS01
RS04
XMU1401
NATL1A
NATL2A
PAC1
MIT0801
MIT0917
MIT0912
MIT0913
MIT0915
MIT1013
MIT1214
SS120
MIT9211
SS35
SS2
SS52
SS51
MIT0602
MIT0601
LG
MIT0603
MIT0918
MIT0919
MIT1304
MIT9303
MIT9313
MIT0702
MIT0703
MIT0701
MIT1303
MIT1320
MIT1205
MIT1227
MIT1418
MIT1306
MIT1312
MIT1323
MIT1327
MIT1342
MIT1318
MIT1313
P1361
P1363
P1344

Fig. S16

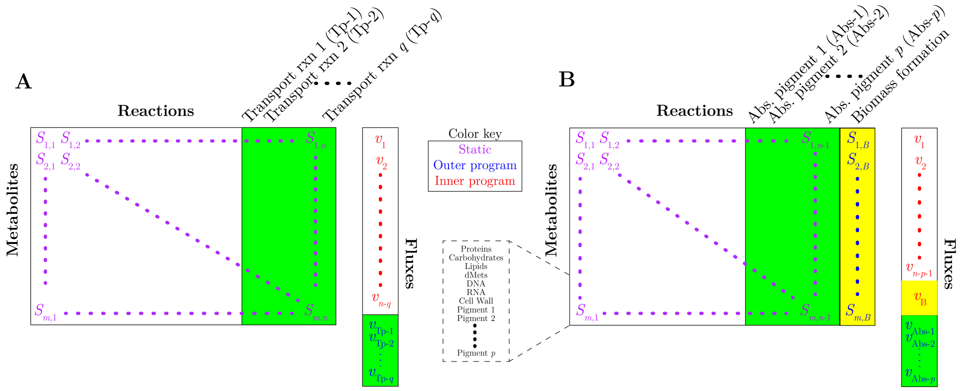
A
Reactions
Metabolites
Fluxes
Transport rxn 1 (Tp-1)
Transport rxn 2 (Tp-2)
Transport rxn q (Tp-q)
Color key
Static
Outer program
Inner program
Proteins
Carbohydrates
Lipids
dMets
DNA
RNA
Cell Wall
Pigment 1
Pigment 2
Pigment p
B
Reactions
Metabolites
Fluxes
Abs. pigment 1 (Abs-1)
Abs. pigment 2 (Abs-2)
Abs. pigment p (Abs-p)
Biomass formation

Fig. S17

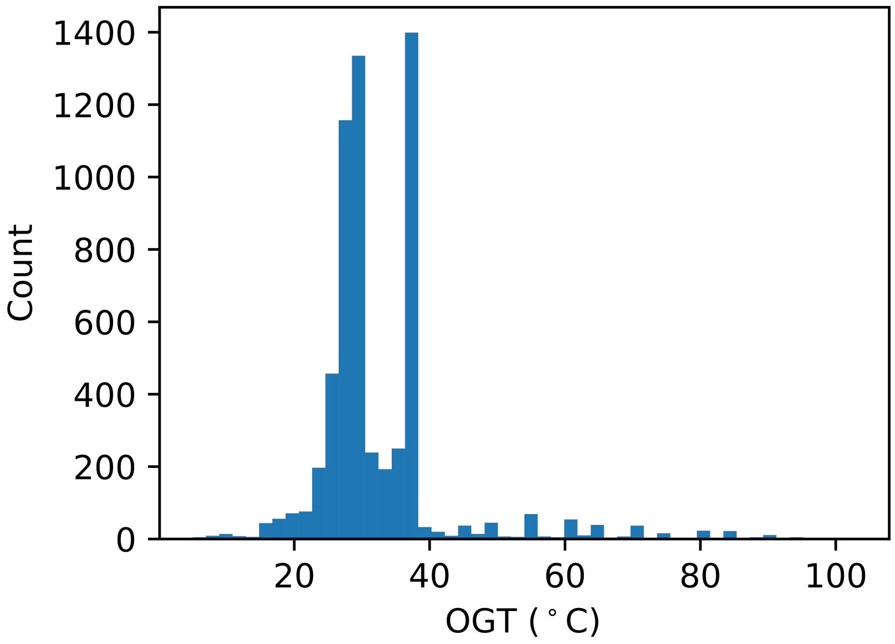

Fig. S18

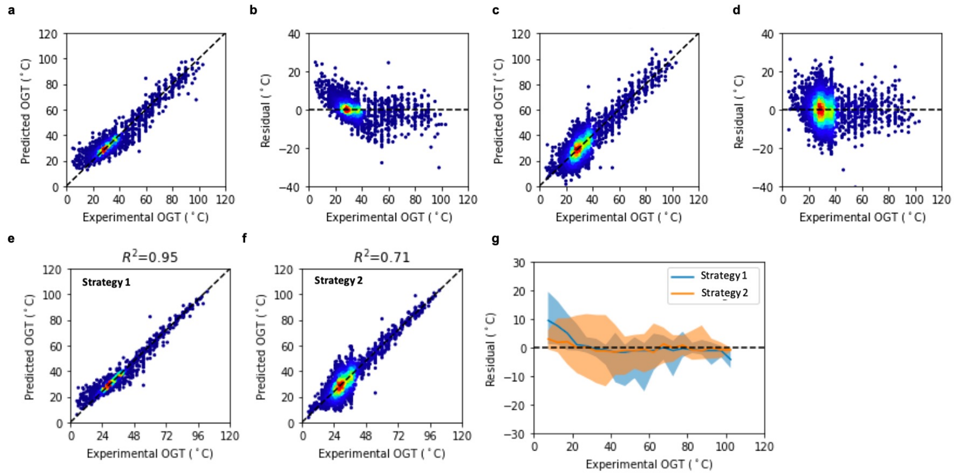

Fig. S19

REFERENCES AND NOTES

1. W. K. Li, Primary production of prochlorophytes, cyanobacteria, and eucaryotic ultraphytoplankton: Measurements from flow cytometric sorting. *Limnol. Oceanogr.* **39**, 169–175 (1994).

2. H. Liu, H. A. Nolla, L. Campbell, *Prochlorococcus* growth rate and contribution to primary production in the equatorial and subtropical North Pacific Ocean. *Aquat. Microb. Ecol.* **12**, 39–47 (1997).

3. Y. M. Rii, S. Duhamel, R. R. Bidigare, D. M. Karl, D. J. Repeta, M. J. Church, Diversity and productivity of photosynthetic picoeukaryotes in biogeochemically distinct regions of the South East Pacific Ocean. *Limnol. Oceanogr.* **61**, 806–824 (2016).

4. F. Partensky, *Prochlorococcus*, a marine photosynthetic prokaryote of global significance. *Microbiol. Mol. Biol. Rev.* **63**, 106–127 (1999).

5. F. Partensky, L. Garczarek, *Prochlorococcus*: Advantages and limits of minimalism. *Ann. Rev. Mar. Sci.* **2**, 305–331 (2010).

6. N. Kashtan, S. E. Roggensack, S. Rodrigue, J. W. Thompson, S. J. Biller, A. Coe, H. Ding, P. Marttinen, R. R. Malmstrom, R. Stocker, M. J. Follows, R. Stepanauskas, S. W. Chisholm, Single-cell genomics reveals hundreds of coexisting subpopulations in wild *Prochlorococcus*. *Science* **344**, 416–420 (2014).

7. P. M. Berube, S. J. Biller, T. Hackl, S. L. Hogle, B. M. Satinsky, J. W. Becker, R. Braakman, S. B. Collins, L. Kelly, J. Berta-Thompson, A. Coe, K. Bergauer, H. A. Bouman, T. J. Browning, D. de Corte, C. Hassler, Y. Hulata, J. E. Jacquot, E. W. Maas, T. Reinthaler, E. Sintes, T. Yokokawa, D. Lindell, R. Stepanauskas, S. W. Chisholm, Single cell genomes of *Prochlorococcus*, *Synechococcus*, and sympatric microbes from diverse marine environments. *Sci. Data* **5**, 180154 (2018).

8. E. R. Zinser, A. Coe, Z. I. Johnson, A. C. Martiny, N. J. Fuller, D. J. Scanlan, S. W. Chisholm, *Prochlorococcus* ecotype abundances in the North Atlantic Ocean as revealed by an improved quantitative PCR method. *Appl. Environ. Microbiol.* **72**, 723–732 (2006).

9. Z. I. Johnson, E. R. Zinser, A. Coe, N. P. McNulty, E. M. S. Woodward, S. W. Chisholm, Niche partitioning among *Prochlorococcus* ecotypes along ocean-scale environmental gradients. *Science* **311**, 1737–1740 (2006).

10. R. R. Malmstrom, A. Coe, G. C. Kettler, A. C. Martiny, J. Frias-Lopez, E. R. Zinser, S. W. Chisholm, Temporal dynamics of *Prochlorococcus* ecotypes in the Atlantic and Pacific oceans. *ISME J.* **4**, 1252–1264 (2010).

11. A. W. Thompson, K. Kouba, N. A. Ahlgren, Niche partitioning of low-light adapted *Prochlorococcus* subecotypes across oceanographic gradients of the North Pacific Subtropical Front. *Limnol. Oceanogr.* 1548–1562 (2021).

12. A. W. Thompson, K. Kouba, Differential activity of coexisting *Prochlorococcus* ecotypes. *Front. Mar. Sci.* **6**, 701 (2019).

13. S. J. Biller, P. M. Berube, J. W. Berta-Thompson, L. Kelly, S. E. Roggensack, L. Awad, K. H. Roache-Johnson, H. Ding, S. J. Giovannoni, G. Rocap, L. R. Moore, S. W. Chisholm, Genomes of diverse isolates of the marine cyanobacterium *Prochlorococcus*. *Sci. Data* **1**, 140034 (2014).

14. T. O. Delmont, A. M. Eren, Linking pangenomes and metagenomes: The *Prochlorococcus* metapangenome. *PeerJ* **6**, e4320 (2018).

15. R. Braakman, M. J. Follows, S. W. Chisholm, Metabolic evolution and the self-organization of ecosystems. *Proc. Natl. Acad. Sci.* **114**, E3091–E3100 (2017).

16. F. Partensky, N. Hoepffner, W. K. W. Li, O. Ulloa, D. Vaulot, Photoacclimation of Prochlorococcus sp. (Prochlorophyta) strains isolated from the North Atlantic and the Mediterranean sea. *Plant Physiol.* **101**, 285–296 (1993).

17. L. R. Moore, S. W. Chisholm, Photophysiology of the marine cyanobacterium *Prochlorococcus*: Ecotypic differences among cultured isolates. *Limnol. Oceanogr.* **44**, 628–638 (1999).

18. M. W. Lomas, J. A. Bonachela, S. A. Levin, A. C. Martiny, Impact of ocean phytoplankton diversity on phosphate uptake. *Proc. Natl. Acad. Sci.* **111**, 17540–17545 (2014).

19. S. Bertilsson, O. Berglund, D. M. Karl, S. W. Chisholm, Elemental composition of marine *Prochlorococcus* and *Synechococcus*: Implications for the ecological stoichiometry of the sea. *Limnol. Oceanogr.* **48**, 1721–1731 (2003).

20. A. C. Martiny, C. T. A. Pham, F. W. Primeau, J. A. Vrugt, J. K. Moore, S. A. Levin, M. W. Lomas, Strong latitudinal patterns in the elemental ratios of marine plankton and organic matter. *Nat. Geosci.* **6**, 279–283 (2013).

21. P. Flombaum, J. L. Gallegos, R. A. Gordillo, J. Rincon, L. L. Zabala, N. Jiao, D. M. Karl, W. K. W. Li, M. W. Lomas, D. Veneziano, C. S. Vera, J. A. Vrugt, A. C. Martiny, Present and future global distributions of the marine cyanobacteria *Prochlorococcus* and *Synechococcus*. *Proc. Natl. Acad. Sci.* **110**, 9824–9829 (2013).

22. P. Lange, R. Brewin, G. Dall'Olmo, G. Tarran, S. Sathyendranath, M. Zubkov, H. Bouman, Scratching beneath the surface: A model to predict the vertical distribution of *Prochlorococcus* using remote sensing. *Remote Sens. (Basel)* **10**, 847 (2018).

23. M. J. Follows, S. Dutkiewicz, S. Grant, S. W. Chisholm, Emergent biogeography of microbial communities in a model ocean. *Science* **315**, 1843–1846 (2007).

24. H. Tettelin, D. Riley, C. Cattuto, D. Medini, Comparative genomics: The bacterial pan-genome. *Curr. Opin. Microbiol.* **11**, 472–477 (2008).

25. C. Lieven, M. E. Beber, B. G. Olivier, F. T. Bergmann, M. Ataman, P. Babaei, J. A. Bartell, L. M. Blank, S. Chauhan, K. Correia, C. Diener, A. Dräger, B. E. Ebert, J. N. Edirisinghe, J. P. Faria, A. M. Feist, G. Fengos, R. M. T. Fleming, B. García-Jiménez, V. Hatzimanikatis, W. van Helvoirt, C. S. Henry, H. Hermjakob, M. J. Herrgård, A. Kaafarani, H. U. Kim, Z.

King, S. Klamt, E. Klipp, J. J. Koehorst, M. König, M. Lakshmanan, D.Y. Lee, S. Y. Lee, S. Lee, N. E. Lewis, F. Liu, H. Ma, D. Machado, R. Mahadevan, P. Maia, A. Mardinoglu, G. L. Medlock, J. M. Monk, J. Nielsen, L. K. Nielsen, J. Nogales, I. Nookaew, B. O. Palsson, J. A. Papin, K. R. Patil, M. Poolman, N. D. Price, O. Resendis-Antonio, A. Richelle, I. Rocha, B. J. Sánchez, P. J. Schaap, R. S. Malik Sheriff, S. Shoaie, N. Sonnenschein, B. Teusink, P. Vilaça, J. O. Vik, J. A. H. Wodke, J. C. Xavier, Q. Yuan, M. Zakhartsev, C. Zhang, MEMOTE for standardized genome-scale metabolic model testing. *Nat. Biotechnol.* **38**, 272–276 (2020).

26. G. Li, K. S. Rabe, J. Nielsen, M. K. M. Engqvist, Machine learning applied to predicting microorganism growth temperatures and enzyme catalytic optima. *ACS Synth. Biol.* **8**, 1411–1420 (2019).

27. D. Vaulot, D. Marie, R. J. Olson, S. W. Chisholm, Growth of *Prochlorococcus*, a photosynthetic prokaryote, in the equatorial Pacific ocean. *Science* **268**, 1480–1482 (1995).

28. C. L. Follett, S. Dutkiewicz, F. Ribalet, E. Zakem, D. Caron, E. V. Armbrust, M. J. Follows, Trophic interactions with heterotrophic bacteria limit the range of *Prochlorococcus*. *Proc. Natl. Acad. Sci. U.S.A.* **119**, e2110993118 (2022).

29. R. W. Eppley, B. J. Peterson, Particulate organic matter flux and planktonic new production in the deep ocean. *Nature* **282**, 677–680 (1979).

30. J. R. Casey, M. W. Lomas, J. Mandecki, D. E. Walker, *Prochlorococcus* contributes to new production in the Sargasso Sea deep chlorophyll maximum. *Geophys. Res. Lett.* **34**, L10604 (2007).

31. R. W. Sterner, J. J. Elser, *Ecological Stoichiometry: The Biology of Elements from Molecules to the Biosphere* (Princeton Univ. Press, 2002).

32. J. R. Casey, M. J. Follows, A steady-state model of microbial acclimation to substrate limitation. *PLOS Comput. Biol.* **16**, e1008140 (2020).

33. R. L. Mather, S. E. Reynolds, G. A. Wolff, R. G. Williams, S. Torres-Valdes, E. M. S. Woodward, A. Landolfi, X. Pan, R. Sanders, E. P. Achterberg, Phosphorus cycling in the North and South Atlantic Ocean subtropical gyres. *Nat. Geosci.* **1**, 439–443 (2008).

34. S. Chávez, J. M. Lucena, J. C. Reyes, F. J. Florencio, P. Candau, The presence of glutamate dehydrogenase is a selective advantage for the cyanobacterium *Synechocystis* sp. strain PCC 6803 under nonexponential growth conditions. *J. Bacteriol.* **181**, 808–813 (1999).

35. O. A. Rangel, G. Gómez-Baena, A. López-Lozano, J. Diez, J. M. García-Fernández, Physiological role and regulation of glutamate dehydrogenase in *Prochlorococcus* sp. strain MIT9313. *Environ. Microbiol. Rep.* **1**, 56–64 (2009).

36. R. W. Sterner, J. J. Elser, E. J. Fee, S. J. Guildford, T. H. Chrzanowski, The light: Nutrient ratio in lakes: The balance of energy and materials affects ecosystem structure and process. *Am. Nat.* **150**, 663–684 (1997).

37. C. Six, Z. V. Finkel, A. J. Irwin, D. A. Campbell, Light variability illuminates niche-partitioning among marine picocyanobacteria. *PLOS ONE* **2**, e1341 (2007).

38. D. Mella-Flores, C. Six, M. Ratin, F. Partensky, C. Boutte, G. L. Corguillé, N. Blot, P. Gourvil, C. Kolowrat, L. Garczarek, D. Marie, *Prochlorococcus* and *Synechococcus* have evolved different adaptive mechanisms to cope with light and UV stress. *Front. Microbiol.* **3**, 285 (2012).

39. C. S. Ting, G. Rocap, J. King, S. W. Chisholm, Cyanobacterial photosynthesis in the oceans: The origins and significance of divergent light-harvesting strategies. *Trends Microbiol.* **10**, 134–142 (2002).

40. C. Steglich, M. Futschik, T. Rector, R. Steen, S. W. Chisholm, Genome-wide analysis of light sensing in *Prochlorococcus*. *J. Bacteriol.* **188**, 7796–7806 (2006).

41. M. Stomp, J. Huisman, L. J. Stal, H. C. P. Matthijs, Colorful niches of phototrophic microorganisms shaped by vibrations of the water molecule. *ISME J.* **1**, 271–282 (2007).

42. H. A. Bouman, T. Platt, M. Doblin, F. G. Figueiras, K. Gudmundsson, H. G. Gudfinnsson, B. Huang, A. Hickman, M. Hiscock, T. Jackson, V. A. Lutz, F. Mélin, F. Rey, P. Pepin, V. Segura, G. H. Tilstone, V. van Dongen-Vogels, S. Sathyendranath, Photosynthesis–irradiance parameters of marine phytoplankton: Synthesis of a global data set. *Earth Syst. Sci. Data* **10**, 251–266 (2018).

43. S. Bertilsson, O. Berglund, M. J. Pullin, S. W. Chisholm, Release of dissolved organic matter by *Prochlorococcus*. *Vie et Milieu* **55**, 225–232 (2005).

44. S. Gudmundsson, I. Thiele, Computationally efficient flux variability analysis. *BMC Bioinform.* **11**, 489 (2010).

45. J. Nogales, S. Gudmundsson, E. M. Knight, B. O. Palsson, I. Thiele, Detailing the optimality of photosynthesis in cyanobacteria through systems biology analysis. *Proc. Natl. Acad. Sci. U.S.A.* **109**, 2678–2683 (2012).

46. H. Knoop, Y. Zilliges, W. Lockau, R. Steuer, The metabolic network of *Synechocystis* sp. PCC 6803: Systemic properties of autotrophic growth. *Plant Physiol.* **154**, 410–422 (2010).

47. S. W. Chisholm, Prochlorococcus. *Curr. Biol.* **27**, R447-R448 (2017).

48. S. Zhang, D. A. Bryant, The tricarboxylic acid cycle in cyanobacteria. *Science* **334**, 1551–1553 (2011).

49. R. Taffs, J. E. Aston, K. Brileya, Z. Jay, C. G. Klatt, S. M. Glynn, N. Mallette, S. Montross, R. Gerlach, W. P. Inskeep, D. M. Ward, R. P. Carlson, In silico approaches to study mass and energy flows in microbial consortia: A syntrophic case study. *BMC Syst. Biol.* **3**, 114 (2009).

50. R. Mahadevan, B. Palsson, D. R. Lovley, In situ to in silico and back: Elucidating the physiology and ecology of *Geobacter* spp. using genome-scale modelling. *Nat. Rev. Microbiol.* **9**, 39–50 (2011).

51. A. R. Zomorrodi, C. D. Maranas, OptCom: A multi-level optimization framework for the metabolic modeling and analysis of microbial communities. *PLOS Comput. Biol.* **8**, e1002363 (2012).

52. O. Perez-Garcia, G. Lear, N. Singhal, Metabolic network modeling of microbial interactions in natural and engineered environmental systems. *Front. Microbiol.* **7**, 673 (2016).

53. J. D. Liefer, A. Garg, M. H. Fyfe, A. J. Irwin, I. Benner, C. M. Brown, M. J. Follows, A. W. Omta, Z. V. Finkel, The macromolecular basis of phytoplankton C:N:P under nitrogen starvation. *Front. Microbiol.* **10**, 763 (2019).

54. L. Heirendt, S. Arreckx, T. Pfau, S. N. Mendoza, A. Richelle, A. Heinken, H. S. Haraldsdóttir, J. Wachowiak, S. M. Keating, V. Vlasov, S. Magnusdóttir, C. Y. Ng, G. Preciat, A. Žagare, S. H. J. Chan, M. K. Aurich, C. M. Clancy, J. Modamio, J. T. Sauls, A. Noronha, A. Bordbar, B. Cousins, D. C. el Assal, L. V. Valcarcel, I. Apaolaza, S. Ghaderi, M. Ahookhosh, M. Ben Guebila, A. Kostromins, N. Sompairac, H. M. le, D. Ma, Y. Sun, L. Wang, J. T. Yurkovich, M. A. P. Oliveira, P. T. Vuong, L. P. el Assal, I. Kuperstein, A. Zinovyev, H. S. Hinton, W. A. Bryant, F. J. Aragón Artacho, F. J. Planes, E. Stalidzans, A. Maass, S. Vempala, M. Hucka, M. A. Saunders, C. D. Maranas, N. E. Lewis, T. Sauter, B. Ø. Palsson, I. Thiele, R. M. T. Fleming, Creation and analysis of biochemical constraint-based models using the COBRA Toolbox v.3.0. *Nat. Protoc.* **14**, 639–702 (2019).

55. A. N. Smith, G. M. M. Hennon, E. R. Zinser, B. C. Calfee, J. W. Chandler, A. D. Barton, Comparing *Prochlorococcus* temperature niches in the lab and across ocean basins. *Limnol. Oceanogr.* **66**, 2632–2647 (2021).

56. E. R. Zinser, Z. I. Johnson, A. Coe, E. Karaca, D. Veneziano, S. W. Chisholm, Influence of light and temperature on *Prochlorococcus* ecotype distributions in the Atlantic Ocean. *Limnol. Oceanogr.* **52**, 2205–2220 (2007).

57. D. A. Ratkowsky, R. K. Lowry, T. A. McMeekin, A. N. Stokes, R. E. Chandler, Model for bacterial culture growth rate throughout the entire biokinetic temperature range. *J. Bacteriol.* **154**, 1222–1226 (1983).

58. J. P. Bowman, in *Psychrophiles: From Biodiversity to Biotechnology: Second Edition*, R. Margesin, Ed. (Springer International Publishing, 2017), pp. 345–387.

59. J. S. Madin, D. A. Nielsen, M. Brbic, R. Corkrey, D. Danko, K. Edwards, M. K. M. Engqvist, N. Fierer, J. L. Geoghegan, M. M. Gillings, N. C. Kyrpides, E. Litchman, C. E. Mason, L. Moore, S. L. Nielsen, I. T. Paulsen, N. D. Price, T. B. K. Reddy, M. A. Richards, E. P. C. Rocha, T. M. Schmidt, H. Shaaban, M. Shukla, F. Supek, S. G. Tetu, S. Vieira-Silva, A. R. Wattam, D. A. Westfall, M. Westoby, A synthesis of bacterial and archaeal phenotypic trait data. *Sci. Data* **7**, 170 (2020).

60. E. Marañón, P. M. Holligan, M. Varela, B. Mouriño, A. J. Bale, Basin-scale variability of phytoplankton biomass, production and growth in the Atlantic Ocean. *Deep Sea Res. I Oceanogr. Res. Pap.* **47**, 825–857 (2000).

61. J. A. Gomez, K. Höffner, P. I. Barton, DFBAlab: A fast and reliable MATLAB code for dynamic flux balance analysis. *BMC Bioinform.* **15**, 409 (2014).

62. S. R. Estévez, Z. Nikoloski, Context-specific metabolic model extraction based on regularized least squares optimization. *PLOS ONE* **10**, e0131875 (2015).

63. B. A. S. Van Mooy, H. F. Fredricks, B. E. Pedler, S. T. Dyhrman, D. M. Karl, M. Koblízek, M. W. Lomas, T. J. Mincer, L. R. Moore, T. Moutin, M. S. Rappé, E. A. Webb, Phytoplankton in the ocean use non-phosphorus lipids in response to phosphorus scarcity. *Nature* **458**, 69–72 (2009).

64. A. Flamholz, E. Noor, A. Bar-Even, R. Milo, eQuilibrator—The biochemical thermodynamics calculator. *Nucleic Acids Res.* **40**, D770–D775 (2012).

65. Y. Zhu, J. E. Graham, M. Ludwig, W. Xiong, R. M. Alvey, G. Shen, D. A. Bryant, Roles of xanthophyll carotenoids in protection against photoinhibition and oxidative stress in the cyanobacterium *Synechococcus* sp. strain PCC 7002. *Arch. Biochem. Biophys.*, 86–99 (2010).

66. I. Thiele, B. Ø. Palsson, A protocol for generating a high-quality genome-scale metabolic reconstruction. *Nat. Protoc.* **5**, 93–121 (2010).

67. H. Wang, S. Marcisauskas, B. J. Sanchez, I. Domenzain, D. Hermansson, R. Agren, J. Nielsen, E. J. Kerkhoven, RAVEN 2.0: A versatile toolbox for metabolic network reconstruction and a case study on *Streptomyces coelicolor*. *PLOS Comput. Biol.* **14**, e1006541 (2018).

68. S. Ofaim, S. Sulheim, E. Almaas, D. Sher, D. Segrè, Dynamic allocation of carbon storage and nutrient-dependent exudation in a revised genome-scale model of *Prochlorococcus*. *Front. Genet.* **12**, 586293 (2021).

69. M. Lynch, G. K. Marinov, The bioenergetic costs of a gene. *Proc. Natl. Acad. Sci. U.S.A.* **112**, 15690–15695 (2015).

70. E. S. Nakayasu, C. D. Nicora, A. C. Sims, K. E. Burnum-Johnson, Y.-M. Kim, J. E. Kyle, M. M. Matzke, A. K. Shukla, R. K. Chu, A. A. Schepmoes, J. M. Jacobs, R. S. Baric, B.-J. Webb-Robertson, R. D. Smith, T. O. Metz, MPLEx: A robust and universal protocol for single-sample integrative proteomic, metabolomic, and lipidomic analyses. *mSystems* **1**, e00043-16 (2016).

71. K. Hiller, J. Hangebrauk, C. Jäger, J. Spura, K. Schreiber, D. Schomburg, MetaboliteDetector: Comprehensive analysis tool for targeted and nontargeted GC/MS based metabolome analysis. *Anal. Chem.* **81**, 3429–3439 (2009).

72. T. Kind, G. Wohlgemuth, D. Y. Lee, Y. Lu, M. Palazoglu, S. Shahbaz, O. Fiehn, FiehnLib: Mass spectral and retention index libraries for metabolomics based on quadrupole and time-of-flight gas chromatography/mass spectrometry. *Anal. Chem.* **81**, 10038–10048 (2009).

73. P. Qian, X.-m. Jiang, Y. Zhang, Analysis of nutritive composition and pigment in *Prochlorococcus*. *J. Ningbo Univ.* **21**, 43–48 (2008).

74. M. Heldal, D. J. Scanlan, S. Norland, F. Thingstad, N. H. Mann, Elemental composition of single cells of various strains of marine *Prochlorococcus* and *Synechococcus* using x-ray microanalysis. *Limnol. Oceanogr.* **48**, 1732–1743 (2003).

75. N. J. Hawco, M. M. McIlvin, R. M. Bundy, A. Tagliabue, T. J. Goepfert, D. M. Moran, L. Valentin-Alvarado, G. R. DiTullio, M. A. Saito, Minimal cobalt metabolism in the marine cyanobacterium *Prochlorococcus*. *Proc. Natl. Acad. Sci. U.S.A.* **117**, 15740–15747 (2020).

76. H. Claustre, A. Bricaud, M. Babin, F. Bruyant, L. Guillou, F. le Gall, D. Marie, F. Partensky, Diel variations in *Prochlorococcus* optical properties. *Limnol. Oceanogr.* **47**, 1637–1647 (2002).

77. D. Marie, F. Rigaut-Jalabert, D. Vaulot, An improved protocol for flow cytometry analysis of phytoplankton cultures and natural samples. *Cytometry A* **85**, 962–968 (2014).

78. L. R. Moore, A. Coe, E. R. Zinser, M. A. Saito, M. B. Sullivan, D. Lindell, K. Frois-Moniz, J. Waterbury, S. W. Chisholm, Culturing the marine cyanobacterium Prochlorococcus. *Limnol. Oceanogr.* **5**, 353 (2007).

79. T. Masuko, A. Minami, N. Iwasaki, T. Majima, S.-I. Nishimura, Y. C. Lee, Carbohydrate analysis by a phenol-sulfuric acid method in microplate format. *Anal. Biochem.* **339**, 69–72 (2005).

80. M. Domínguez-Martín, G. Gómez-Baena, J. Díez, M. J. López-Grueso, R. J. Beynon, J. M. García-Fernández, Quantitative proteomics shows extensive remodeling induced by nitrogen limitation in *Prochlorococcus marinus* SS120. *mSystems* **2**, 1 (2017).

81. M. Källberg, H. Wang, S. Wang, J. Peng, Z. Wang, H. Lu, J. Xu, Template-based protein structure modeling using the RaptorX web server. *Nat. Protoc.* **7**, 1511–1522 (2012).

82. W. Hayduk, H. Laudie, Prediction of diffusion coefficients for nonelectrolytes in dilute aqueous solutions. *AIChE J.* **20**, 611–615 (1974).

83. J. Arnold, Studies in diffusion. II. A kinetic theory of diffusion in liquid systems. *J. Am. Chem. Soc.* **52**, 3937–3955 (1930).

84. A. Bricaud, H. Claustre, J. Ras, K. Oubelkheir, Natural variability of phytoplanktonic absorption in oceanic waters: Influence of the size structure of algal populations. *J. Geophys. Res. Oceans* **109**, C11010 (2004).

85. P. J. B. Williams, N. W. Jenkinson, A transportable microprocessor-controlled precise Winkler titration suitable for field station and shipboard use. *Limnol. Oceanogr.* **27**, 576–584 (1982).

86. Y. M. Kim, B. J. Schmidt, A. S. Kidwai, M. B. Jones, B. L. Deatherage Kaiser, H. M. Brewer, H. D. Mitchell, B. O. Palsson, J. E. McDermott, F. Heffron, R. D. Smith, S. N. Peterson, C. Ansong, D. R. Hyduke, T. O. Metz, J. N. Adkins, Salmonella modulates metabolism during growth under conditions that induce expression of virulence genes. *Mol. Biosyst.* **9**, 1522–1534 (2013).
